# Exploring Mental Health Services for Youth Experiencing Homelessness in East Asian Pacific Regions: A Systematic Scoping Review

**DOI:** 10.3390/children11070864

**Published:** 2024-07-17

**Authors:** Kimberley Cortez Ermita, Diana Margot Rosenthal

**Affiliations:** 1 UCL Great Ormond Street Institute of Child Health, University College London, London WC1N 1EH, UK; kimberley.ermita.22@alumni.ucl.ac.uk; 2 UCL Collaborative Centre for Inclusion Health, University College London, London WC1E 7HB, UK; 3 Department of Social and Behavioral Sciences, New York University School of Global Public Health, New York, NY 10003, USA

**Keywords:** youth experiencing homelessness, East Asia Pacific, mental health interventions, cognitive behavioural therapy, inclusion health, resilience enhancement, life skills education, government programs, emotional health

## Abstract

Background: Youth experiencing homelessness (YEH) in East Asian Pacific (EAP) regions represent one of the most at-risk populations due to cultural and geographical factors. Effective mental health interventions, primarily researched in Western contexts, may not fully apply to YEH in EAP. Their lack of stable shelter, disrupted social networks, and limited access to mental health services elevate their susceptibility to adverse mental health, making urgent interventions essential to address their needs. Objective: The objective of this study is to explore and systematically search the types of mental health services and interventions available for YEH in EAP and their impact on overall quality of life and wellbeing. Methods: Electronic databases (e.g., Medline, PsycINFO, PubMed, Scopus) were systematically searched (publication dates between 1 January 1990 and 13 May 2023), as well as additional online resources specific to homelessness. Articles were screened, and a critical appraisal assessed the quality of the included studies. Results: Eight studies with different interventions were identified in Indonesia (*n* = 2), Malaysia (*n* = 1), South Korea (*n* = 3), and the Philippines (*n* = 1). These were thematically clustered into six categories: art, cognitive behavioural therapy, life skills education, resilience enhancement, family strengthening, and government interventions/services. Conclusions: This review highlights effective mental health interventions’ positive impact on YEH mental health outcomes and quality of life in EAP, stressing the urgent need to implement socio-culturally sensitive services. Future research should address knowledge gaps through comprehensive studies covering diverse EAP regions and populations, prioritising socio-culturally specific psychological measures.

## 1. Introduction

### 1.1. Background

Youth experiencing homelessness (YEH) is a serious global public health concern—they represent one of the most at-risk populations in many countries worldwide [1]. Globally, 1.6 billion people are estimated to experience some form of inadequate housing [2]; however, the true prevalence of homelessness is unknown [3]. In East Asian Pacific (EAP) regions, youth homelessness demands urgent attention. Although there is not a single unifying cause found to elucidate routes that lead youth to homelessness, poverty plays a significant role in exacerbating homelessness in EAP regions [4,5]. EAP suffers the most significant damage caused by natural hazards (e.g., earthquakes, tsunamis, tropical cyclones/typhoons) [6] that are often neglected by the state during recovery and rebuilding periods [7]. As such, economic inequality has been shown to exacerbate stress within families, which can manifest negative outcomes; these include selling or abandoning their children [5,8] or family dysfunction, such as domestic violence and abuse, which are considered major forces that push children and young people (CYPs) to self-migrate, leave home, or run away [9,10], and therefore, they are often left to fend for themselves.

YEH are at high risk for various adverse mental health outcomes [1]. They experience abuse and neglect, both prior to leaving home and while living on the streets (i.e., rough sleeping), that often lead to significant, long-lasting trauma [11]. For instance, high rates of depression, posttraumatic stress disorder (PTSD), substance abuse, suicidal ideation, and bipolar disorder have been reported among YEH [12,13]. YEH are faced with multiple daily stressors associated with street life and often demonstrate a lack of coping strategies, resilience and essential social skills needed to sustain social support [14,15,16,17,18]. Therefore, they are likely to resort to maladaptive coping strategies [19] and experience decreased quality of life compared with the general population [20]. Notably, suicidality is the leading cause of death among YEH [21], and high rates of suicide attempts have been reported among this population (80%) [22]. Furthermore, previous research documented that YEH were highly susceptible to becoming targets of sexual exploitation and prostitution [23], even among sheltered youth [24]. While the mentioned studies were valuable in shedding light on the risks associated with YEH, they were all conducted in Western countries. This observation underscores a gap in the existing literature, which could potentially leave an incomplete understanding of the broader issue of homelessness and its associated risks in other parts of the world.

### 1.2. Problems of Defining ‘Homelessness’

Defining ‘homelessness’ is complex: There is no internationally agreed definition as it plays out along a continuum [1,3]. Finding overall figures on YEH in EAP is extremely challenging given the different local and government definitions of homelessness from each country in this region, along with the absence of reliable data [1,3]. The most commonly used term to refer to YEH in the academic literature and policymakers in EAP regions (e.g., China and Indonesia) is ‘*street children*’, defining those under the age of 18 years who live or work in public areas with little or no parental supervision [25,26]. ‘*Runaway youths*’ is another term used mainly in Korean literature [27,28], along with ‘*street sleeping*’, ‘*rough sleeping*’, and ‘*pavement dwelling*’ to describe primary homelessness [29,30]. CYPs experiencing secondary homelessness, defined by their lack of stable long-term housing, frequently between temporary accommodations such as shelters, orphanages, and refugee camps [3,30]. ‘*Invisible homelessness*’ is a phenomenon observed in many EAP countries, where young people couch-surf between friends’ homes, 24 h cafés, or fast-food restaurants due to cultural norms and societal pressures [31], making their housing instability less visible but equally pressing.

Different government definitions of homelessness vary across border lines. For example, in Singapore, ‘homelessness’ is defined as “*any person found begging in a public place in such a way as to cause or be likely to cause annoyance to persons frequenting the place or otherwise to create a nuisance*” [32], whereas in South Korea, it is defined as “*persons who have used/lived homeless facilities for a substantial period*” [33]. As highlighted by Rosenthal et al. (2021), the varying definitions of homelessness can have significant implications for the provision of mental health services depending on how it is recognised within a given jurisdiction [34] and, therefore, could act as a barrier to accessing mental health services. There is a lack of government policies that mention mental health services for this population based on those definitions [25], implying that mental health services are not adequately addressed by the government.

### 1.3. Challenges of YEH in EAP and Rationale for the Review

YEH in EAP are a distinct population and are influenced by a combination of cultural, socioeconomic, and contextual factors. While both the West and East grapple with the challenges of youth homelessness, the unique dynamics of the EAP regions amplify the importance of research in this area. For instance, cultural norms and values, along with rapid urbanisation and migration, might contribute to different pathways to homelessness [3,5]. Barriers to accessing mental health services may differ from those in Western countries. Some examples were, but are not limited to, low perceived need [35], rural and financial constraints [36,37], and a lack of mental health professionals and resources who only practice in urban areas [38,39,40]. These factors can reinforce stigmatisation and marginalisation in countries where there is a strong societal stigma and shame already attached to homelessness [41,42]. The associated risks and effective mental health interventions aforementioned for this population were experiences of YEH in Western countries with Western therapeutic systems. These experiences and risks may differ for Asian adolescents experiencing homelessness living in EAP, as an extensive body of literature places emphasis on the different perceptions of mental illness cross-culturally [43,44,45,46], which then can, in turn, influence both their receptiveness and adherence to treatment plans. Understanding these cross-cultural differences is crucial for mental health professionals to provide effective and culturally sensitive care for YEH, as it involves adapting treatment strategies that align with cultural norms [47] in EAP. Although several studies have evaluated the effectiveness of interventions for this population [48,49,50], there is a lack of understanding of the different mental health interventions and programs for youth homelessness in EAP regions. Most systematic reviews and previous studies have focused on interventions conducted primarily in Western countries [51,52,53]. Yet, to date, there is a lack of a comprehensive overview of the literature surrounding interventions and programs for youth homelessness in EAP alone.

To address the research gap in the international literature, the primary objective of this review was to systematically search and provide evidence on the different types of mental health services and interventions for YEH in EAP and their impact on overall quality of life and wellbeing for YEH. Thus, a scoping review [54,55] was selected to help map out the existing literature on this topic by gaining a comprehensive understanding of the current state of such services and interventions for YEH in the region. This review can hopefully provide valuable insights for future research, policy formulation, and program development that is tailored to this distinct and at-risk population in order to improve the quality of life and positive health outcomes for YEH in EAP regions.

## 2. Methods

This review followed the Centre for Evidence-Based Management (CEBMa) for Critically Appraised Topics guidelines [54] and PRISMA Extension for Scoping Reviews checklist [55] (Table S1) to ensure that the review process was conducted in a comprehensive manner and to minimise potential researcher bias [56]. The research question was formulated using the PICO (population, intervention, comparison, and outcome) framework [57]. Table 1 presents the full inclusion and exclusion criteria with definitions.

### 2.1. Databases and Search Strategy

The following major electronic databases were searched for this review: Medline, PsycINFO, PubMed, Scopus, and Web of Science. Additional records were hand-searched through other sources, such as web-based publications and grey literature specific to homelessness, reference lists of included texts, and related publications. These included Centre for Homelessness Impact (CHI) [60], a non-profit organisation dedicated to providing evidence-based solutions and innovations with the goal of improving the outcomes of people experiencing homelessness. The search was conducted on 13th May 2023 and was limited and filtered to English records published between 1990 and 2023 in all databases. Table 2 presents an example of search terms used for Medline, with slight variations employed in each database. See Table S2 for full search terms used in all academic databases. A prior search was conducted across official government websites to compare different definitions of homelessness within EAP regions and to inform the search terms used (Table 2 and Table S2).

### 2.2. Screening Process

**Figure 1:**
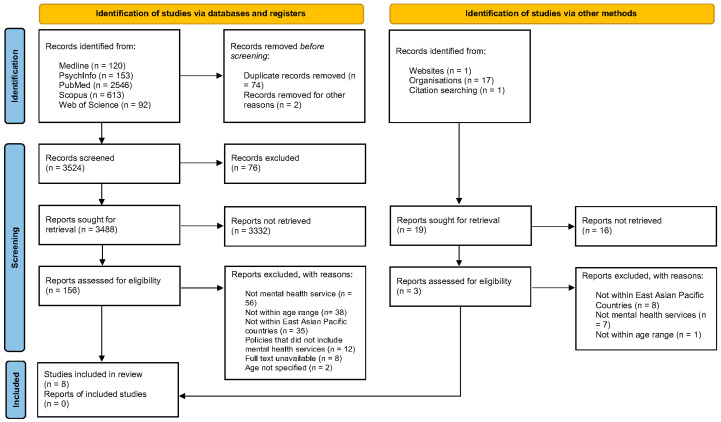
PRISMA 2020 flow diagram [61] for new systematic reviews, which included searches of databases, registers, and other sources.

Results were imported into EndNote 2.0 for screening and deduplication. An initial title and abstract screening was conducted. Full-text articles were then read and thoroughly evaluated to determine eligibility for inclusion in the systematic scoping review. Studies meeting the inclusion criteria were selected, and subsequent data extraction was carried out. Each study was independently reviewed by two coders, KCE and DMR. Any conflicts were discussed in depth between the co-authors until a consensus was reached. For example, through consensus meetings for both the screening and data extraction stages, the coders referred back to the inclusion criteria to ensure consistency. This rigorous process ultimately led to achieving 100% interrater reliability (IRR) for the included articles in this review. This is demonstrated in the PRISMA flow diagram [61] in Figure 1.

### 2.3. Analysis

A study quality assessment was performed using the Critical Appraisal Skills Programme (CASP) checklists [62,63,64,65] (see Supplementary Materials S3 for the CASP checklist that was matched and employed for each selected text and its study design). Each selected study was evaluated to assess its methodological robustness, validity, and relevance to the research question. Applicability to clinical practice and ability to generalise results were also assessed, which was crucial for this review. This ensured that the evaluated interventions and programs were relevant for addressing specific mental health needs of YEH. CASP [62,63,64,65] aims to inform evidence-based practice as generalisability helps identify interventions that have demonstrated their effectiveness across diverse settings for the target population [66]. Lastly, interventions/programs in the results were thematically grouped based on the type of interventions they encompassed.

## 3. Results

### 3.1. Study Selection

A total of 3543 publications were identified from the literature search across all databases and through hand-searching additional resources, which represented 3467 publications after duplicates were removed and screened for eligibility. After the selection process, a total of eight studies published between 2000 and 2023 met the inclusion criteria and were included in this review. Figure 1 is the PRISMA flow diagram [61] showing individual database numbers and articles retrieved.

### 3.2. Study Characteristics

All included articles were conducted within the EAP regions: Indonesia (*n* = 2) [67,68], Malaysia (*n* = 1) [69], South Korea (*n* = 3) [27,70,71], and the Philippines (*n* = 1) [72]. The location of one study (*n* = 1) was withheld for confidentiality reasons, but it was conducted in Southeast Asia [73], e.g., Cambodia, Indonesia, Laos, Malaysia, Myanmar, Philippines, Thailand, and Vietnam [74,75], indicating it still fit the inclusion criteria. Outreach settings for YEH primarily consisted of participants residing in orphanages (*n* = 8), shelters (*n* = 8), centres (*n* = 4), and children residing on the street (*n* = 2).

#### 3.2.1. Participant Characteristics

The age range of the participants varied across the studies, with the majority falling between the inclusion criteria of ages 12 to 18 yrs. (*n* = 4) [27,69,71,72]. However, a case study included participants as young as 7 years old [67], with the oldest participants in another study being 21 [70]. Both male and female participants were included in the studies; some studies focused exclusively on one gender to examine gender-specific effects of interventions for YEH [27,70], while others had a balanced representation of both genders [69]. A summary of the study and participant characteristics is reflected in Table 3. As the selected studies were conducted within the EAP regions, the studies encompassed a diverse range of participant ethnic and religious backgrounds, such as Christianity and Islam. In the Malaysian study [69], there were different ethnicities included as participants, although they only reflected a small percentage. For example, a large percentage of participants in this study were of Malay ethnicity (67.5%), followed by Indian (21.8%), Chinese (4.8%), and Indonesian and Orang Asli [Indigenous] (5.9%). Lastly, a large majority of participants across all eight studies belonged to families of low socioeconomic position (SEP) (Table 3).

#### 3.2.2. Study Designs

Of the eight studies, three involved some type of randomised control trial (RCT) study design. One of these included a parallel single-blind (subject-masked) RCT [69]; one study had randomly assigned participants to either a control group or intervention group [27]; the other study was a development of an intervention program for YEH, with the aims of using an RCT to evaluate its effectiveness of the developed program [71]. Four studies used quasi-experimental and qualitative designs [68,70,72,73], and the remaining one was a case study [67] (Table 3). Each selected study was subjected to quality checks using the CASP checklists [62,63,64,65] matched by study design (see Supplementary Materials S3).

#### 3.2.3. Outcome Measures

A structured overview of measures and outcomes for each selected study is displayed in Table 4. Evidently, a range of outcome measures were utilised to evaluate various aspects of the mental wellbeing and health status of YEH. For example, depression was measured with different scales across the studies: Brillantes-Evangelista [72] used the Self-Rating Depression Scale (SDS) [76,77], while Hyun et al. [27] and Mohammadzadeh et al. [69] used alternative scales to measure depression (Beck Depression Inventory [BDI] and Depression, Anxiety, and Stress Scales [DASS-21]; respectively) [78,79]. For specific outcome measures, the choice of instruments varied across studies, which were *usually* translated into the country’s main language (Filipino, Malay, or Korean).

### 3.3. Mental Health Interventions/Programs

This review identified eight unique mental health programs and interventions for YEH in EAP. These were clustered thematically into six categories:

1. **Art**: interventions that provide a creative and non-verbal outlet for self-expression, healing, and personal growth for YEH [72,73];
2. **Cognitive behavioural therapy**: an intervention that targets negative emotional states and cognitive distortions by developing coping strategies [27];
3. **Life skills education:** interventions that equip YEH with essential practical abilities in the real world [69];
4. **Resilience enhancement**: an intervention to improve protective factors associated with resilience [70];
5. **Family strengthening**: interventions that explicitly target families in the program as a key focus that fosters positive relationships and support networks [71];
6. **Government interventions/services**: the role of the government in supporting YEH [67,68].

Each selected study was organised by thematic category below and subjected to quality checks using the CASP checklists [62,63,64,65] matched by study design (see Supplementary Materials S3).

**Art.** Two studies used a quasi-experimental design to evaluate the effectiveness of different forms of art as a therapeutic intervention for abused youth in orphanages and militarised children in centres, which took place in the Philippines [72] and in Southeast Asia [73]. Art as an intervention for these studies included art psychotherapy, poetry psychotherapy, and art without boundaries as a general communication and therapeutic tool.

The objectives of these studies were to evaluate the effectiveness of visual arts and poetry as interventions to alleviate depression and PTSD symptomatology [72], as well as to understand the children’s perceptions of hope [73]. Although each study reported on different measures of art as an intervention, both studies reported themes of art creating a sense of agency, catharsis, and personal empowerment for YEH. In one study, Brillantes-Evangelista (2013) [72] measured PTSD and depression symptoms using various instruments (see Table 4), including self-reported scales and participant observation throughout the intervention period. Results showed a decrease in depressive symptoms in mean scores from pre-test to post-test among the group receiving poetry psychotherapy, indicating a positive impact on their wellbeing (Table 4), whereas a reduction of PTSD symptoms in mean scores was evident among the visual arts group, suggesting that this form of art therapy was beneficial for addressing trauma-related symptoms for YEH [72]. Brillantes-Evangelista [72] noted that although these results suggest that visual arts and poetry psychotherapy were effective in reducing psychological symptoms for abused adolescents living in orphanages, art seemed to have been helpful in other ways by empowering YEH to actively engage in their own healing and recovery. For instance, poetry allows youth to find meaning and purpose in their experiences by transforming hardships into something meaningful and artistic that can inspire hope. The notion of ‘hope’ was also noted in the second study [73], where militarised children visualised their aspirations, dreams, and personal goals through art. Miles (2000) [73] concluded that despite their circumstances, art had allowed them to envision a positive future and work towards achieving it, instilling hope for a better tomorrow.

**CBT.** One RCT study conducted by Hyun, Chung, and Lee (2005) in South Korea evaluated the effects of group CBT on self-esteem, depression, and self-efficacy of runaway adolescents residing in shelters [27]. The authors measured these using Western-derived scales (Table 4) that were translated into Korean and employed in pre- and post-tests. In this study, the most common reason for running away was parental abuse, especially by their father (40.7%), and family conflict (29.6%). There were also high rates of reported problem drinking (66.7%) and smoking (77.8%) among YEH. Results demonstrated a reduction in depression and improved self-efficacy in the experimental group from pre-test to post-test, while there were no significant changes in documented self-esteem pre- and post-tests for both experimental and control groups (see Table 4). However, the authors revealed that an eight-week intervention period may be too short to yield noticeable results in self-esteem [27]. The study’s RCT design and use of validated measures (e.g., BDI [78]; Table 4) enhanced the reliability of the study’s findings (CASP [64]; Supplementary Materials S3) [62]. However, the geographical limitations should be considered when applying the results to YEH populations in different regions in EAP (see Table 5 for the full strengths and limitations of each study).

**Life skills education.** Mohammadzadeh et al. (2017) [69] conducted a parallel single-blind (subject-masked) RCT in Malaysia, which evaluated the effects of life skills-based intervention programs on the emotional health and self-esteem of adolescents residing in orphanages [69]. This program was designed based on the life skills education (LSE) formulated by the World Health Organisation (WHO) [96]—a structured, evidence-based guideline for children and adolescents stemming from the stress-coping theory by Lazarus and Folkman [97]. The LSE framework was a valuable resource for YEH, as it equipped them with essential skills and abilities to effectively navigate challenges they experienced in everyday life, promoting personal growth and resilience [96]. The study’s intervention sessions focused on a range of life skills: self-awareness, critical and creative thinking, communication, intra- and interpersonal relationships, problem-solving, decision-making, empathy, and coping with emotion and stress. Outcomes were assessed using DASS-21 [79] and a Malay version of Rosenberg’s [86] Self-Esteem Scale (RSES) [87] at pre-test, post-test, and four-month follow-up (F/U). Results revealed that the LSE program significantly decreased mean scores for anxiety, depression, and stress and increased self-esteem from pre-test to post-test for participants receiving intervention. Notably, there was an increase in depression scores from the post-test to the four-month F/U, implying that LSE demonstrated efficacy in addressing depression, yet its impact was insufficient for sustainable change beyond the four-month period (Table 4). These findings [69] could have high potential for research in different localities within EAP regions because of their good replicability and RCT design, especially because this study had a diverse range of ethnicities. However, an important consideration was that the intervention period was not specified (Table 5).

**Resilience enhancement.** A study on Korean female runaway adolescents by Noh (2018) assessed the efficacy of a resilience enhancement program using a quasi-experimental design [70]. This design was appropriate based on ethical considerations and real-world settings, as it allowed the researcher to observe the program in an orphanage without compromising ethical guidelines or manipulating variables (CASP [64]; Supplementary Materials S3). The resilience enhancement program was developed based on interviews with female runaway adolescents residing in shelters and a comprehensive review of the literature focused on individual protective factors relevant to YEH and runaway adolescents. Resilience, depression, anxiety, and problem drinking were assessed using self-reported questionnaires (Table 4) at pre-test, post-test, and at a one-month F/U. While the intervention group exhibited notable reductions in depression at pre-test and at a one-month F/U, this decrease was not exclusive to the overall study period: Depressive symptoms decreased over the study duration for both the intervention and control groups. Reductions in anxiety levels were also observed in the intervention group compared with the control, and interestingly, the control group experienced a rise in anxiety levels at the one-month F/U [70]. In terms of problem drinking, the average levels decreased consistently for the intervention group throughout the three time points (Table 4). Although these were preliminary results, the small p-value suggested that the program had an effect on the dependent variables that were unlikely to occur by random chance alone [70].

**Family strengthening.** Noh and Choi (2020) developed a family-based mental health program for runaway adolescents residing in youth shelters using an intervention mapping (IM) protocol [71]. Despite its efficacy and effectiveness not yet being evaluated, this program focused on family strengthening that aligned with Korean cultural values that shaped their context. It was developed by conducting a comprehensive literature review and interviews with runaway adolescents and shelter workers. A logical framework outlining family relationships and mental health issues faced by runaway adolescents was then developed based on the problems that were identified during the preliminary IM process. The program used theory- and evidence-based methods for practical applications such as motivational interviewing, cognitive reappraisal, consciousness-raising, skills training, guided practice, social modelling, improving emotional states, and verbal persuasion. Runaway adolescents receiving the intervention will have a total of eight individual and family sessions altogether. The family sessions consisted of four themes: family engagement and establishing motivation for change, rebuilding relationships with family, improving family communication, and improving collaborative problem-solving. The effectiveness of the developed program will be evaluated using an RCT on adolescents residing in shelters aged 12 to 18 [71]. Data will be collected using self-reported questionnaires.

The developed family-based mental health program has high study potential for YEH specific to the EAP context. However, to date, this program has not been tested for its efficacy and effectiveness and might not yield the positive health outcomes expected from the authors.

**Government interventions/services.** The following two papers [67,68] focused on government interventions/programs and/or policies regarding the mental health of YEH, thereby meeting the inclusion criteria (Table 1).

One research case study paper addressed the policies implemented by the Makassar City Region in Indonesia in managing street children by collecting primary data (field observations and key informants) and secondary data (official government documents and literature publications on street children) [68]. They identified three social services implemented by the Makassar City Region in managing street children (Table 6).

Despite these service policies, Solong et al. (2023) [68] identified factors inhibiting government policy in managing street children. These included insufficient quantity of human resources, budget limitations, insufficient facilities and infrastructure, an unstable community economy, and a lack of strict implementations for regional regulations regarding the management of street children. In spite of that, initial steps have been carried out by the government to prevent the development and expansion of street children. These included collaboration with Civil Service Police Units to patrol street children’s activities and/or remove them from the street environment and into police stations so that they can be then placed into orphanages [68].

The Kampung Anak Negeri (KAN) was a children’s village established by the Surabaya City Government in Indonesia that protected the rights of street children, aiming to empower street children with the goal of granting them agency to enhance their self-resilience to deter them from returning to the street. Sarmini and Sukartiningsih (2018) evaluated the role of the KAN in facilitating the transition of street children towards a regular life [67]. Data were gathered by participatory observation and in-depth interviews with the street children placed in the KAN. The KAN established by the Surabaya City Government had six roles and responsibilities [67]. These responsibilities are described in Table 7.

Overall, KAN serves as a comprehensive support system for street children that respects and integrates Indonesian culture into its program, aiming to improve mental health outcomes and ultimately break the cycle of street life and create a better future for themselves [67].

## 4. Discussion

The main objective of this review was to systematically search and provide evidence on the different types of mental health services and interventions for YEH in EAP and their impact on the overall quality of life and wellbeing of YEH. A wide variety of mental health interventions and programs for YEH in EAP was identified. These included art and poetry psychotherapy for abused Filipino adolescents living in shelters [72], art without boundaries for militarised children [73], CBT for runaway Korean adolescents [27], a life skills education program for Malaysian adolescents living in orphanages [69], a resilience enhancement program for female runaway Korean adolescents [70], and development of a family-based mental health program for runaway adolescents [71]. In essence, these mental health interventions and programs were effective in alleviating symptoms of anxiety, depression, stress, and PTSD, as well as targeting problem drinking. Additionally, there were notable increases in overall resilience and self-efficacy for YEH in EAP, and they were taught various life skills to tackle any daily stressors they may experience.

Notably, there were only two programs and social services found in this review that were established and implemented by the government that targeted street children [67,68]. While the researchers did not directly assess the outcomes of these services, their reports underscored the importance of creating culturally sensitive programs empowering and improving the quality of life for YEH in Indonesia, as well as identifying factors inhibiting the effective implementation of government policies aimed at addressing the needs and challenges faced by street children [67,68].

Prior to this review, most systematic reviews and previous studies had mainly focused on interventions conducted in Western countries. Furthermore, there was a lack of comprehensive overview of the literature surrounding interventions and programs for YEH in EAP alone, although there was an extensive body of literature emphasising the different perceptions of mental illness cross-culturally, which may impact the acceptability of treatment options and adherence to treatment plans [43]. In this review, risks associated with youth homelessness in EAP, such as stress, maladaptive behaviour, and suicidal behaviours, were reportedly similar compared to Western countries (e.g., Hyun et al. [27] and Moskowitz et al. [19], respectively). Additionally, consistent with findings from Western literature [11,13], baseline assessments also revealed high levels of adverse mental health outcomes in various studies conducted in EAP [27,69,70,72]. Nonetheless, Western-developed therapeutic systems such as CBT and LSE were also found to be effective in alleviating symptoms in Korean and Malay adolescents [27,69].

Having said that, it is essential to highlight that these instruments and measures were socio-culturally specific, which could have influenced the efficacy and effectiveness of treatment response. For example, five studies translated the measures into the country’s main language [27,69,70,72,73]. Translating the psychological instruments into the country’s language was crucial, given the nature of the targeted population for this review. YEH may have limited proficiency in the English language, especially if most of the participants belong to families of low SEP and were residing in orphanages, shelters or on the street and have not completed or received formal education [27,67,68,69,70,71,72,73]. Although this was not specified in the studies, YEH with limited education may lead to feelings of discomfort or shame when responding to instruments in a language they were unfamiliar with. Therefore, using their first language may have reduced stigma and encouraged them to share their experiences openly, promoting accessibility and inclusivity in the research process for this population [98].

### 4.1. Exploring Mental Health Interventions and Programs

Six key themes were identified among the eight included studies: art, CBT, LSE, resilience enhancement, family strengthening, and government interventions/services.

#### 4.1.1. Art

Art-based interventions utilise creative mediums that do not primarily rely on spoken language to convey emotions, thoughts and experiences [99]. This has been well documented in numerous past and recent Western studies with adolescents in bridging that gap between therapists and adolescents [100,101]. It uses a child-centred approach [102]. As demonstrated in a sample of militarised children in SE Asia [73], it breaks down communication barriers and acts as a facilitator between the researcher and the CYPs [73]. This was especially beneficial for YEH, as art can be used as a tool to amplify their voices in a society where it is marginalised and unheard [99]. This, in turn, may reduce the overall stigma surrounding homelessness, shifting the focus from defining youth homelessness solely by their housing status to appreciating their artistic talents and creativity; it helps people see their multifaceted identities beyond homelessness, as shown in the Philippines [72]. Yet, it is important to note these findings may not be generalised to youth who have learning disabilities (LDs) and/or cognitive difficulties in engaging in complex art or poetry activities. Even so, with tailored interventions and adaptations, art and poetry psychotherapy can minimise barriers and promote autonomy, which empowers YEH with LDs to enhance their self-expression and build confidence, wherein traditional therapeutic approaches can lack [103].

#### 4.1.2. CBT

Findings from South Korea [27] have been reported in the previous literature by Rohde et al. [104] for adolescents with comorbid substance abuse and in parallel with more recent findings for street children in Mexico City [105] and in Iran [106]. The effectiveness of cognitive techniques in CBT for reconstructing positive worldviews was notable as it identified and challenged negative thought patterns [107]. Importantly, the two themes in Hyun et al.’s study, ‘*developing coping strategies*’ and ‘*planning for future life*’ [27], equipped runaway youths with practical coping skills suited to their circumstance. For example, it encouraged them to think long-term beyond immediate survival needs, thus promoting a sense of direction and pathways to stability such as housing, employment, and education. Additionally, improved self-efficacy, indicated by the theme ‘*raising self-consciousness*’, aligned with Bandura’s [108] model of self-efficacy. In the context of homelessness, this model postulated that with the appropriate training, runaway adolescents could interpret emotional and physical reactions (e.g., anxiety and stress) as signs of competency, which increased their overall self-efficacy, evident in this study [108].

Although results from this study demonstrated that CBT had no effect on self-esteem, this has been challenged by a recent meta-analysis that found CBT-based interventions to be efficacious for treating low self-esteem [109]. Yet, these findings may not be generalisable to Korean adolescents, as the analysis was conducted on adults in the UK, where self-esteem is prioritised in Western cultures [110]. In contrast, self-esteem is conceptualised differently, as shown elsewhere in the East, among a more comparable sample of Chinese children [111]. Self-esteem is multifaceted and complex, influenced by a myriad of internal and external factors; it could simply be that the intervention period of eight weeks was too short to yield a noticeable impact on self-esteem [27]. Short intervention periods may not adequately address cultural nuances and beliefs that impact how self-esteem is perceived and influenced in Korean runaway adolescents [43]. Additionally, it should be highlighted that the subjects of this study were all male, and there could be potential gender disparities in self-efficacy, depression, self-esteem presentations, and responsiveness to CBT [27]. Thus, future research could benefit in this area by exploring the long-term effects of culturally specific and extended interventions to develop more comprehensive and effective interventions that address unique challenges for this vulnerable population.

#### 4.1.3. LSE

LSE for depressive symptoms was found insufficient for sustainable change [69]. This observation could be attributed to the inherent characteristics of youth homelessness. The lack of continued support that they had received during the intervention period may lead to a resurgence of depressive symptoms, given that depression is a highly recurrent disorder [112]. In other words, if the adolescent returned to the same challenging or unstable living conditions or environment after the intervention, the stressors associated with homelessness could be precipitating factors for the re-emergence of depressive symptoms. It appeared LSE had a more sustainable effect on anxiety, stress, and self-esteem [69]. Comparable outcomes have been observed in previous studies among Indian adolescents [113] and, more recently, in Indonesian students [114].

In contrast, LSE illustrated positive effects on self-esteem and resilience for YEH living in orphanages [69]. This phenomenon could be explained by the intricate interplay between life skills development and positive feelings of self-worth. Learning and mastering certain life skills can contribute to a positive self-perception and higher self-esteem from feelings of accomplishment [115], and conversely, having positive self-worth can motivate them to develop and practice their life skills [113]. As YEH develop practical skills, their self-worth and confidence are likely to improve, which, in turn, can motivate them to further enhance their life skills and actively engage in efforts to overcome their challenges. Ultimately, this positive cycle can empower this population by equipping them with valuable tools and a stronger sense of self, fostering resilience and assisting their journey towards stability and self-sufficiency.

#### 4.1.4. Resilience Enhancement

Resilient enhancement programs [70] offer significant advantages for runaway adolescents, as these can positively influence mental wellbeing and appear promising for enhancing resilience and mitigating problem drinking. Factors associated with resilience, such as self-esteem and self-regulation, have been well documented in the previous literature with Korean adults [18,116], while relational and problem-solving skills [117] and goal-setting skills [118] have been considered protective factors for runaway adolescents. Since the intervention program used in Noh’s study [70] integrated the abovementioned protective factors linked to resilience, these elements were believed to have contributed to the observed increase in resilience among participants in the intervention group [70].

In terms of explaining reduced anxiety, Noh’s study [70] provided progressive relaxation training and deep breathing techniques that counteracted physiological responses to stress [119]. These equipped YEH with the immediate skills to manage moments of crisis and distress, which, therefore, increased their overall resilience [120]. The issue of youth homelessness is often interconnected with the challenge of problem drinking in both the East and the West, as YEH may resort to alcohol as a coping mechanism to navigate the hardships and uncertainties they encounter on the streets [121,122]. The current finding of decreased problem drinking was consistent with previous findings in the UK by Rew et al. [123]; both Noh [70] and Rew et al. [123] included components of assertive and effective communication, as well as goal-setting skills in their interventions, which are recognised as protective factors in building resilience. In essence, resilience enhancement programs hold the promise of catalysing positive transformation in the lives of female runaway adolescents by fostering their growth [70].

#### 4.1.5. Family Strengthening

The role of the family in addressing runaway behaviour is important as it holds paramount importance not only in Korean culture but also in many EAP cultures [71]. Although psychological interventions for youth homelessness have been evaluated in previous Western studies, the authors suggested that these interventions do not reflect the actual situation Korean runaway adolescents experience [71]. Thus, the family-based mental health program Noh and Choi [71] developed aimed to improve mental health outcomes for this at-risk population using socio-cultural contexts. During their literature review, the authors identified that runaway adolescents experience high rates of dissatisfaction with family life [124]. Additionally, family factors such as poor communication and emotional support at home, financial problems, and poor psychological wellbeing of the parent or caregiver [125] can affect mental health and quality of life. As a result, the runaway adolescents who participated in the interviews revealed they were more likely to engage in risky and maladaptive behaviours as a response to stress from family problems [125]. The identified issues have been supported by a range of parent–child theories. For example, the parental monitoring theory [126] emphasised the importance of parental supervision and awareness of adolescents’ activities. If the runaway adolescent experienced a lack of parental warmth because of their poor communication and emotional support, they were more likely to be involved in risky behaviour associated with homelessness/running away. As the developed program recognised the cultural significance of family in Korean society, it emphasised the importance of re-establishing strong family connections to deter adolescents from engaging in runaway behaviour [71]. Ultimately, the parent–child collaboration aligned with the cultural fabric of Korean society and aimed to create a nurturing environment where adolescents feel supported and understood without resorting to running away. Understanding the parent–child dynamic can offer valuable insights for other EAP societies facing similar challenges. It underscores the significance of familial bonds, open communication, and community support structures to provide a safety net for YEH. This emphasises a proactive intervention in addressing the root causes of youth homelessness, offering effective strategies that prioritise prevention and holistic support.

### 4.2. Government Interventions/Services

Interestingly, while both EAP and Western countries aim to address youth homelessness, there are notable differences in their government interventions/services due to varying cultural, social, and economic contexts. For example, while Western programs often emphasise individual autonomy and direct service provision [127], government interventions/services in EAP regions may place greater emphasis on familial or community support networks that implement spiritual values [67]. Moreover, there exists a more established network of government-funded health and wellbeing services in the West, encompassing mental health and substance abuse counselling [128]. In contrast, the selected studies in this review revealed that access to healthcare services in EAP regions was more limited, with fewer specialised services for YEH provided by the government. In summary, tailoring support specific to socio-cultural contexts is crucial for effectively addressing the unique needs of YEH, and future efforts should focus on bridging these gaps to ensure equitable access to comprehensive support services for this vulnerable population in EAP.

### 4.3. Limitations

To the best of our knowledge, there have been no previous systematic or scoping reviews on the different types of mental health services and interventions for YEH in EAP and their impact on the overall quality of life and wellbeing for YEH. Despite finding positive mental health outcomes for YEH resulting from these psychological interventions and programs, there were several limitations of this review that should be noted. First, one of the primary objectives of this review was to identify different mental health interventions and programs for youth homelessness in EAP. According to The World Bank [58], there are 15 countries within EAP, and yet, this review only found evidence of effective interventions and government programs in four named countries (i.e., Indonesia, Malaysia, South Korea, the Philippines) with the majority being conducted in South Korea (*n* = 3). Importantly, seven (87.5%) of the reviewed studies were conducted in urban areas or in the capital city, which might not be reflective of the broader population. Homelessness patterns might differ significantly between urban and rural settings. Factors contributing to homelessness, available resources, programs or interventions, and support networks can be different in various parts of a country, especially if many of the EAP cultures rely on traditional folk medicine and healers [129].

Second, subpopulations of youth homelessness, such as LGBTQ+ or Indigenous youth, might be more prevalent in certain regions or outside the capital city. Focusing solely on urban areas could also neglect the unique challenges faced by these groups. Additionally, the only variation of ‘homelessness’ that was not included in this review was ‘*invisible homelessness*’ as there was no existing literature that met this review’s inclusion criteria. Third, differential definitions of homelessness used by the studies and the data that were collected do not make them comparable. However, considering shared characteristics and successes of similar programs in both Western and Eastern countries, this might not have been a significant factor contributing to the effectiveness of Western therapies, suggesting that tailoring and adaption could enhance their efficacy. The differences in age of YEH and sampling may have affected comparability and generalisability as some YEH were mixed with higher age groups, and some studies only reported the mean age for the sample. Therefore, these settings and groups may not be comparable.

Despite these limitations, a notable aspect where this review excels was that it was not limited to one study design and high-quality methodologies (e.g., RCTs, quasi-experimental design), but it also included case studies for in-depth qualitative data, enabling a more comprehensive and nuanced understanding of youth homelessness in EAP regions. Additionally, six papers (75%) out of the eight selected for this review were all conducted and published relatively recently, ensuring that the findings provided were accurate and relevant to the current mental health interventions, policies, and outcomes for YEH in EAP.

### 4.4. Implications for Future Research, Policy, and Practice

The findings of this review suggest that different mental health interventions and programs have positive impacts on overall mental health outcomes and quality of life for YEH. Despite that, a large gap in robust evidence-based research in the EAP regions is still evident. Future research should prioritise filling these substantial knowledge gaps by conducting comprehensive studies that encompass various EAP regions and diverse populations like YEH. There should be a pressing need to engage in co-designing research and programs with YEH to authentically incorporate lived experience voices. For example, two included studies in this review effectively integrated YEH voices [70,71], underscoring the importance of this approach in comprehending cultural nuances. This inclusive methodology fosters a deeper understanding of the diverse challenges faced by YEH and can enhance the relevance and effectiveness of interventions or programs aimed at supporting them.

Importantly, researchers should strive for inclusive sampling that covers urban, suburban, and rural areas to provide a holistic understanding of unique challenges faced by YEH in different settings. Providing robust research across different EAP regions could provide comparative studies that highlight disparities, commonalities, and explanations for the different pathways that lead to homelessness. This holds particular significance in EAP regions for youth homelessness when contrasted with Western studies, as it helps avoid making assumptions and generalisations that might not hold true across cultural boundaries. For instance, many of the comparative studies were compared with Western studies (such as Bandura et al. [124]; Kolubinski et al.) [109] that do not underpin cultural nuances and local dynamics such as family structures and social norms that are important EAP cultures [71]. Mixed-method studies may be useful for exploring this by combining qualitative and quantitative research techniques to understand the corresponding data while checking the validity of statistical findings [130]. In addition, although some type of RCT study design was used in three studies, there were various limitations, e.g., the intervention period was not specified [69,70,72], too short [27,72], or yet to be conducted [71]. Robust longitudinal analysis should also be implemented to track current situations and identify trends and changes.

Subpopulations, such as LGBTQ+ youth who experience homelessness, also present a critical avenue for gaining insights into the intersection of cultural and identity dynamics in EAP regions, particularly if these dynamics contribute to pathways to homelessness. This subgroup is a very at-risk population in both the West [131] and the East [132], signifying an urgency for research. For example, Confucianism is deeply ingrained in some EAP societies that can shape perceptions of family, community, gender [133], and perhaps even perceptions of homelessness. From a political perspective, LGBTQ+ youths represent the fastest-growing demographic within South Korea’s homeless population [134]. They face exclusion from certain homeless shelters, thus being deprived of human rights protections and social benefits [134]. Future research working with this subgroup of youth homelessness could identify the potential multi-level barriers these youth encounter when seeking assistance. Not only will it contribute to a more comprehensive understanding of the lived experiences of LGBTQ+ youth who experience homelessness, but it could also reduce marginalisation for an already marginalised group in a society where homelessness is heavily stigmatized [41,42].

The scarcity of published literature (such as government services/programs) concerning the management and policies for youth homelessness in EAP underscores the significant gap in addressing this issue. The lack of documented programs reflects a limited institutional response to the challenges faced by this at-risk population, possibly resulting from varying levels of awareness, available resources, and prioritisation [68]. Nonetheless, the findings of positive mental health, health outcomes and quality of life from mental health interventions and programs for YEH in EAP provide valuable insight for policymakers, which has global relevance to ensure that the sustainable development goals [135] are met for 2030. For instance, evidence-based policies can be developed that are rooted in the specific lived experiences and realities of EAP regions. These could potentially include root causes of youth homelessness, such as family conflict [27], or economic factors, such as being at risk of homelessness [68]. Thus, addressing these potential root causes can allow for a prioritisation of preventative measures.

Lastly, this review emphasises the importance of implementing culturally sensitive services adaptable to the diverse cultural contexts within EAP regions, such as translating psychological instruments and measures into the country’s language [27,69,70,72,73] or understanding religious values that are deeply rooted in EAP societies [67,68]. Some implications for practice should highlight the importance of collaborative learning for practitioners dealing with YEH. This fosters a more comprehensive approach to acknowledging the multifaceted challenges these youth experience, which can, in turn, enhance the quality and impact of interventions for this population.

## 5. Conclusions

Despite the positive mental health outcomes found in this review, a lack of established evidence-based interventions/programs and the existing research gap in EAP underscores the urgent need for comprehensive efforts. A strength of this review lies in its emphasis on tailoring and adapting interventions and programs to suit the socio-cultural contexts of EAP regions, and this review demonstrates its potential to inform future interventions and policy decisions. Tailoring and adapting interventions and programs for socio-cultural contexts can also pave the way for improved services and holistic solutions that address the multifaceted challenges faced by these vulnerable populations. In conclusion, while small steps have been made towards devising effective interventions, programs, and policies, it is evident that there is still significant progress to be made.

## Figures and Tables

**Table 1:** Definitions, inclusion, and exclusion criteria.

	Inclusion Criteria	Exclusion Criteria
**P**opulation	□ Studies should have explicitly targeted youth ages 12 to 18 who were experiencing homelessness, previously experienced homelessness, or were currently residing in temporary shelters and orphanages. Although these ages were ideal, some room for margin could be adjusted if it met the rest of the inclusion criteria or if the mean age was between the ages of 12 and 18; □ This review was not limited to the term “homeless”, but “street children”, “children of the street”, “street youth”, “runaway adolescents/youth”, and “refugee children and youth” were also considered; □ Moving forward, in this review, ‘*homelessness*’ was defined according to aforementioned terms street children, runaway youths, and adolescents residing in temporary shelters and were used interchangeably; □ Studies must have been conducted in EAP regions that were in accordance with the World Bank’s list at the time of search: Cambodia, China, Indonesia, Korea, Lao PDR, Malaysia, Mongolia, Myanmar, Papua New Guinea, Pacific Islands, Philippines, Singapore, Thailand, Timor-Leste, and Vietnam [58,59].	□ Adolescents not within the age range; □ Adolescents who had not previously experienced/were not currently experiencing homelessness; □ Studies that were not conducted within EAP regions;
**I**ntervention	□ This review included any mental health intervention, and programs that targeted the study population were eligible for inclusion in this review. Examples of mental health interventions are, but are not limited to, cognitive behavioural therapy (CBT), family-based therapy, and therapeutic support. See Table 2 for a full list of search terms used for mental health interventions.	□ Government policies/reports/services/programs that do not include mental health services and interventions.
**C**omparison	□ No specific comparisons were made for this review.	
**O**utcome	□ The primary outcome of this review was to explore different mental health interventions for YEH in EAP regions.	

**Table 2:** Example search terms. Search terms used for Medline.

Category	Search Terms
YEH	((homeless* and (child* or youth* or adolescen* or teen* or young person* or young people*)) or street child* or street sleep* or “homeless* youth” or ill-housed person* or rough sleeper* or railway boy* or street dweller* or refugee*) (Homeless persons or Homelessness or Homeless family or Homeless Shelters or Homeless Youth or Homeless single person or “outreach to the homeless” or homeless mentally ill or homeless shelter resident or Homeless Health Concerns) (Runaways or Runaway children or Street Youth)
AND	
Mental health intervention	(mental health service* or therapeutic support* or counselling or counseling or housing program* or temporary shelter* or homeless shelter* or psychological counseling or psychological counselling or short-term temporary care or short-term care or youth homeless* shelter or non government* organisation* or non government* organization* or non-government* organisation* or non-government* organization* or NGO* or mental health care or mental health support* or cognitive behavioural therap* or cognitive behavioral therap* or CBT* or substance abuse therap* or outreach program* or outreach support* or mental health intervention* or mental health* or life counseling or life counselling or overcrowded or refugee* or emergency accommodation* or homeless* facilit* or rehabilitation* or prevention approach* or social work* or therap*) Mental Health Services/or Child Guidance/or Community Mental Health Services/or Counseling/or Emergency Services, Psychiatric/or Social Work, Psychiatric/ (health service, mental or health services, mental or hygiene service, mental or hygiene services, mental or mental health service or mental health services or mental hygiene service or mental hygiene services or service, mental health or service, mental hygiene or services, mental health or services, mental hygiene)
AND	
EAP countries	(east asia* pacific or east asia* pacific countr* or cambodia* or china or chinese* or hong kong or indonesia* or japan* or south korea* or lao* pdr or macau or macanese or malaysia* or mongolia* or myanmar or pacific island* or papua new guinea or papuans or philippin* or filipin* or the philippine* or singapore* or taiwan* or thai or timor-leste or vietnam*) exp Cambodia/or exp Indochina/or exp Indonesia/or exp Laos/or exp Malaysia/or exp Myanmar/or exp Philippines/or exp Singapore/or exp Thailand/or exp Timor-Leste/or exp Vietnam/or exp China/or exp Japan/or exp Korea/or exp Mongolia/or exp Taiwan/or exp Indonesia/or exp Japan/or exp Macau/or exp Philippines/or exp Taiwan/

Note: The symbol “*” that follows a search term instructs the database to search for any words that share the same stem as the search term—for example, using “adolescen*” would retrieve results containing variations such as “adolescents”, “adolescent”, “adolescence”, etc.

**Table 3:** Summary of study characteristics.

Author(s) and Year	Title	Country of Study	Design	Outreach Setting	Sample Size *n*	Age Range in Years	Gender
Brillantes-Evangelista (2013) [72]	An evaluation of visual arts and poetry as therapeutic interventions with abused adolescents	The Philippines	Quasi-experimental and qualitative methodologies	5 shelters around Metro Manila	33	13 to 18	21 females; 12 males
Hyun et al. (2005) [27]	The effect of Cognitive-Behavioural group Therapy on the self-esteem, depression, and self-efficacy of runaway adolescents in a shelter in South Korea	South Korea	RCT	1 shelter in Seoul	27	Mean age 15.5	27 males
Miles (2000) [73]	Drawing together hope: ‘listening’ to militarised children	SE Asia *	Quasi-experimental	2 centres	60	9 to 16	52 males; 8 females
Mohammadzadeh et al. (2019) [69]	Improving emotional health and self-esteem of Malaysian adolescents living in orphanages through the Life Skills Education program: A multi-centred randomised control trial	Malaysia	Parallel single-blind (subject-masked) RCT	8 orphanages in Klang Valley	271	12 to 18 (mean age 14.47)	149 males; 122 females
Noh (2018) [70]	The Effect of a Resilience Enhancement Program for female runaway youths: A quasi-experimental study	South Korea	Quasi-experimental	5 shelters	32	12 to 21 (mean age 16.69)	Females
Noh & Choi (2020) [71] **	Development of the Family-Based Mental Health Program for runaway adolescents using an intervention mapping protocol	South Korea	RCT	2 youth centres	N/A	Targets 12 to 18 **	N/A
Sarmini & Sukartiningsih (2018) [67]	From road to the arena: The role of Kampung Anak Negeri for street children	Indonesia	Case study	Kampung Anak Negeri (KAN)	N/S	7 to 18	N/S
Solong et al. (2023) [68]	Street child management policy at social office of Makassar City, Indonesia	Indonesia	Qualitative	Social services, street	7 ***	N/S	N/S

N/A: not applicable; N/S: not specified. * Location confidential. ** Program development; no RCT conducted yet. *** 5 informants from social services, 2 street children who received services from the Makassar City Social Service office.

**Table 4:** Mental health services/programs and outcomes for youth experiencing homelessness.

Author(s)	Intervention/Program	Control	Instruments/Measure and Statistical Analysis	Outcome
Brillantes-Evangelista [72]	Art psychotherapy Poetry psychotherapy	**(1)** Visual arts group (*n* = 11) **(2)** Poetry group (*n* = 11) **(3)** Control group (*n* = 11)	□ Self-Rating Depression Scale (SDS) [76,77]; □ Child Report on Posttraumatic Symptoms (CROPS) [76,80]; □ These were translated into Filipino and back-translated into English. SDS and CROPS were administered pre- and post-test. Mid-assessment was employed to detect changes in scores; □ Repeated measures *t*-test was conducted to determine significant differences between pre-test, mid-test (for the intervention groups only), and post-test scores for all 3 groups; □ Participants’ experiences were examined by looking at their behaviours, artwork, and interview responses throughout the intervention period. These were subject to content analysis.	*Statistical outcomes for PTSD symptoms* □ Downward trend on PTSD symptomology pre- and post-test CROPS scores for visual arts (27.72, 23.90, respectively) and poetry groups (27.36, 24.27) compared with control (29.27, 30.71); □ Significant decrease in mean CROPS repeated measure *t*-test scores from pre-test to post-test for visual arts group (*t* (10) = 2.702, *p* = 0.011 **, *r* = 0.6496), which was also evident mid-test (*t* (10) = 2.044, *p* = 0.034 *, *r* = 0.2947); □ General decrease in CROPS scores for poetry group pre-test and post-test (*t* (10) = 1.731, *p* = 0.057), with slight increase from pre- to mid-test (*t* (10) = −0.051, *p* = 0.48); □ Significant decrease in mean scores from pre-test to post-test on CROPS with moderate effect size (*t* (10) = 2.232, *p* = 0.025, *r* = 0.5761). *Statistical outcomes for depression symptoms* □ Decreases in pre-test and post-test scores for all groups, with the poetry group having the largest decrease in scores (43.636 and 39.909, respectively), followed by visual arts (43.545, 40.545) and the control group (44.354; 42.857); □ Significant decrease in mean SDS repeated measures *t*-test scores from pre-test to post-test for poetry group with moderate effect size (*t* (10) = 1.880, *p* = 0.0445 *, *r* = 0.512); □ No significant difference between pre-test, mid-test, and post-test mean scores in the visual arts and control groups. * *p* < 0.05. ** *p* < 0.01.
Hyun et al. [27]	Cognitive behavioural therapy	**(1)** Experimental group (*n* = 14) **(2)** Control group (*n* = 13)	□ Self-esteem inventory [81] translated into Korean [82]; □ Beck Depression Inventory (BDI) [78] translated into Korean [83]; □ Self-efficacy scale [84] translated into Korean [85] was employed pre- and post-test; □ Fisher’s exact probability and the Mann–Whitney *U* test were used to test the homogeneity between the experimental group and the control group in terms of demographics and baseline values (self-esteem, depression, self-efficacy); □ Wilcoxon signed-rank test was used to examine the effects of CBT on self-esteem, depression, and self-efficacy.	No significant differences in homogeneity in the levels of self-esteem, depression, and self-efficacy between the experimental group and control group. *Statistical outcomes for the effect of CBT after intervention* *Self-efficacy* □ Self-efficacy scores increased pre-test (*M* = 53.86, *SD* = 7.65) to post-test (*M* = 60.29, *SD* = 8.08) for experimental group (*z* = −2.098, *p* = 0.36); □ No significant changes for pre-test scores *M* = 45.15, *SD* = 10.57) to post-test (*M* = 47.15, *SD* = 9.47) for control (*z* = −0.969, *p* = 0.333). *Depression* □ Significant decrease in depression pre-test scores (*M* = 15.43, *SD* = 8.42) to post-test scores (*M* = 9.64, *SD* = 8.76) for experimental group (*z* = −2.325, *p* = 0.020); □ Depression occurred after the intervention period (*M* = 15.08 (*SD* = 6.60); *M* = 17.46 (*SD* = 12.57)) for control group (*z* = −0.420, *p* = 0.674). *Self-esteem* □ No significant differences in self-esteem scores pre-test and post-test (*M* = 51.57 (*SD* = 6.96); *M* = 53.86 (*SD* = 10.23)) for experimental group (*z* = −1.191, *p* = 0.234); □ No significant differences in self-esteem scores pre-test and post-test (*M* = 47.62 (*SD* = 6.40); *M* = 50.69 (*SD* = 7.38)) for control group (*z* = −1.691, *p* = 0.091).
Miles [73]	Art	N/A	□ Drawings; □ Children were individually asked to explain what they had drawn and were also discussed with key staff.	Children were individually asked to explain what they had drawn, which was also discussed with key staff. □ 24 refugee boys indicated that they had been ‘soldiers’ in their past lives (usually through holding guns or wearing uniforms); 22 boys drew themselves as soldiers in the future; 13 boys drew themselves as ‘something else’; □ Other children drew themselves as farmers/buffalo herders in the past; □ Some of the children drew themselves as teachers (*n* = 9), preachers/evangelists (*n* = 7), and other jobs (e.g., doctors, politicians);
Mohammadzadeh et al. [69]	Life skills education program	**(1)** Intervention group (*n* = 139) **(2)** Placebo group (*n* = 132)	□ Depression Anxiety Stress Scales (DASS-21 [79]; □ Rosenberg Self-Esteem Scale (RSES) [86] Malay version [87] employed pre- and post-test and at a 4-month follow-up; □ A mixed within- and between-subject ANOVA was used to assess the means differences of the scale variables in the intervention and control groups.	*Intervention effects* □ Significant difference in the mean scores for depression (*f* = 33.80, *p* < 0.001, η^2^ = 0.11) among the 3 time points: pre- and post-test and at a 4-month follow-up; □ Mean scores were significantly different between groups for anxiety (*f* = 6.28, *p* < 0.01, η^2^ = 0.02), stress (*f* = 32.05, *p* < 0.001, η^2^ = 0.11), and self-esteem (*f* = 54.68, *p* < 0.001, η^2^ = 0.17); □ No significant difference between intervention group and placebo group for depression (*f* = 2.33, *p* = 0.13). *Post Hoc (Bonferroni) test (between groups)* □ Differences in depression (Δ mean = −1.72, *p* < 0.001), anxiety (Δ mean = −0.99, *p* < 0.01), stress (Δ mean = −1.97, *p* < 0.001) and self-esteem (Δ mean = 5.24, *p* < 0.001) scores between intervention and control groups at post-test; □ Significant changes in the mean scores for anxiety (Δ mean = −1.92, *p* < 0.001), stress (Δ mean = −3.01, *p* < 0.001) and self-esteem (Δ mean = 4.39, *p* < 0.001) at a 4-month follow-up; □ No significant differences between the intervention and control groups at 4-month follow-up (Δ mean = −0.18, *p* < 0.67). *Post hoc (Bonferroni) test (within groups)* □ Significant difference between pre-test and post-test (Δ mean = 2.00, *p* < 0.001) for depression, stress (Δ mean = 2.80, *p* < 0.001), and self-esteem (Δ mean = −5.48, *p* < 0.001), with a large effect and effect size in the intervention group (η^2^ = 0.32, η^2^ = 0.30, η^2^ = 0.20; respectively); □ No significant difference in the mean scores for the study variables between post-test and follow-up at *p* < 0.001 value except for depression (Δ mean = −1.37, *p* < 0.001) in the intervention group; □ No significant difference was found in the mean scores for depression, anxiety, stress, and self-esteem at *p* < 0.001 value between pre- and post-test and at a 4-month follow-up for control group.
Noh [70]	Resilience enhancement program	**(1)** Experimental group (*n* = 16) **(2)** Control group (*n* = 16)	Self-reported questionnaires on the following: □ The family APGAR [88] as translated into Korean [89] to assess YEH’s perspectives on their family functions; □ Resilience (the Youth Korea Resilience Quotient-27 [YKRQ-27]) [90]; □ Depression (BDI-II) [91] translated into Korean [92]; □ Anxiety (Beck Anxiety Inventory [BAI]) [93] translated into Korean [94]; □ Problem drinking (the Alcohol Use Disorders Identification Test Alcohol Consumption Questions [AUDIT-C]) [95] assessed at pre-test, post-test, and at a one-month follow-up (1 m F/U); □ Mann–Whitney *U* test and Fisher’s exact probability were used to test the homogeneity between experimental and control groups in terms of general characteristics and baseline (pre-test) scores.	No statistically significant difference in homogeneity in general characteristics between experimental group and control group was found. *Resilience* □ Significant group-by-time interaction effects between pre-test and post-test (*β* = 12.42, *p* = 0.002) and at a 1 m F/U (*β* = 12.72, *p* = 0.007); □ Significant increase in resilience was found at baseline (*M* = 80.43, *SD* = 17.86) to post-test (*M* = 91.00, *SD* = 16.27) and at a 1 m F/U (*M* = 87.46, *SD* = 16.27) for experimental group; □ Decreases in resilience for control group across intervention period (*M* = 93.56 (*SD* = 16.49); *M* = 93.00 (*SD* = 16.14); *M* = 89.38 (*SD* = 14.67)). *Depression* □ Significant group-by-time interactions were seen between pre-test and post-test (*β* = −5.33, *p* = 0.037) but not between pre-test and at a 1 m F/U (*β* = −4.48, *p* = 0.120); □ Significant time effect between pre-test and at a 1 m F/U (*β* = −3.33, *p* = 0.030); □ Significant decrease in depression across intervention period for experimental group (pre-test: *M* = 22.00 (*SD* = 13.66); post-test: *M* = 17.00 (*SD* = 15.22); 1 m F/U: *M* = 15.62 (*SD* = 16.08)) and for control group (*M* = 15.00 (*SD* = 10.45); *M* = 12.23 (*SD* = 9.11); *M* = 9.23 (*SD* = 9.93)). *Anxiety* □ Significant group-by-time interaction was seen between pre-test and at a 1 m F/U (*β* = −8.00, *p* = 0.022); □ Average levels of anxiety decreased across the study period for experimental group (pre-test: *M* = 22.56 (*SD* = 13.66); post-test: *M* = 17.00 (*SD* = 15.22); 1 m F/U: *M* = 15.62 (*SD* = 16.08)); □ Decrease in anxiety scores pre-test to post-test for control group (*M* = 7.37 (*SD* = 6.26); *M* = 5.23 (*SD* = 6.52)), but an increase in anxiety was found at 1 m F/U (*M* = 8.23 (*SD* = 12.71)). *Problem drinking* □ Significant group-by-time interaction was seen between pre-test and post-test (*β* = 3.58, *p <* 0.001) and at 1 m F/U (*β* = 0.63, *p* = 0.038); □ Average level of problem drinking decreased across the study period for experimental group (pre-test: *M* = 3.50 (*SD* = 4.10); post-test: *M* = 2.57 (*SD* = 3.82); 1 m F/U: *M* = 1.92 (*SD* = 2.78)); □ Problem drinking scores remain average at post-test for control group (*M* = 2.69 (*SD* = 2.95)) and at a 1 m F/U (*M* = 2.54 (*SD* = 3.18)), which were higher at pre-test (*M* = 2.50 (*SD* = 3.18)).
Noh & Choi [71] †	Family-based mental health program	**(1)** Experimental group **(2)** Comparison group	□ Self-reported questionnaire surveys at baseline, immediately after, and at a 1-month follow-up.	N/A
Sarmini & Sukartiningsih [67]	Kampung Anak Negeri (KAN)	N/A	□ Field observations and in-depth interviews.	KAN was established by the Surabaya City Government to provide social services. The main goals of KAN are to provide education and protection for street children and to return them to their respective families. KAN has 6 responsibilities: □ The needs of street children are the responsibility of the government; □ Educational rights; □ ‘*Pak Ustadz*’, a spiritual adviser; □ Honest, disciplined, and responsible: simple steps to build self-integrity (fulfilling the needs of mental behavioural guidance through coaching); □ Networks as social capital and survival strategies (skills guidance); □ Achievement culture: changing the future orientation of street children.
Solong et al. [68]	N/A	N/A	□ Primary data: field observations and in-depth interviews; □ Secondary data: documents obtained via government agencies on street children, publications (books, journals, magazines).	Three modes of services for street children, according to the Ministry of Social Affairs: 1. *Community-based social services* □ Prevent children from poor families from becoming street children. 2. *Street-based social services* □ Prevent street children from becoming criminals. 3. *Centre-based social services* □ Children are taken from the street and placed in special service institutions such as orphanages. Social services collaborate with several units to prevent/manage street children. □ Collaboration with Civil Service Police to patrol street children’s activities; □ Additionally, children found on the street will be taken to the police station and then taken to an orphanage as their new place of residence; □ Mental, physical, social, and skills guidance during rehabilitation.

N/A: not applicable. † This is a program that has been developed but has not yet been tested for its effectiveness. Control and measure comments are based on how it will be evaluated if the program proceeds in the future.

**Table 5:** Strengths and limitations of each paper.

Author(s) and Year	Strengths	Limitations
Brillantes-Evangelista [72]	□ Considered cultural art practices that were ingrained in Filipino history; □ Measures were translated into Filipino and back-translated into English; □ Visual arts and poetry (VAP) psychotherapy breaks the barrier and transgresses beyond spoken language between therapists/practitioners and participants; □ Good generalisability for cross-cultural research; quasi-experimental design; □ As participants learn the skills of VAP, these skills can be used outside of therapeutic practices and in their daily lives to alleviate depression/PTSD symptomology.	□ Findings cannot be generalised to youth with learning disabilities; □ The main facilitators were not certified art or poetry therapists. These disciplines are not yet institutionalised in the Philippines; □ Confounding variables such as personality and preference (towards visual art or poetry) could have affected participants’ responsiveness to treatment; □ Intervention period was too short (eight sessions); □ Intervention period (in weeks) was not documented; □ Authors did not assess long-term outcomes of depressive/PTSD symptomology.
Hyun et al. [27]	□ Good replicability; RCT design; supported existing literature; □ Clear definition of runaway adolescents; □ Culturally sensitive; measures were translated into Korean; □ Authors reported runaway reasons, which is good for future research on preventing runaway behaviour.	□ Intervention period may be too short to yield noticeable results on self-esteem; □ Age range of participants was not reported—only mean age was reported; □ All participants were male; □ Self-reported inventory—potential under or overreported symptoms; □ Fairly outdated research findings; □ Small sample size.
Miles [73]	□ Child-centred approach; □ Art acts like a facilitator between the researcher and the child; implications on how art is a gateway to the child’s mind; □ Conducted on militarised children, a distinct and at-risk population.	□ Does not specify how drawing helped militarised children psychologically; □ Children’s age will affect what they draw; □ Only the children themselves can understand what they have drawn. The study relies on adult interpretation of the drawings and is subjective; □ Interpreters were the children’s teachers who were also refugees and may have affected the children’s drawings.
Mohammadzadeh et al. [69]	□ Based on WHO’s life skills education (WHO, 1997); □ Covers a range of practical life skills, which is crucial for YEH; □ Large sample size of both male and female participants; □ Measures were translated into Malay; □ Good replicability; RCT design; □ Diverse participant nationality; □ Cost-effective and easy to administer; □ Can help policymakers in paying special attention to life skills-based interventions; □ High study potential for different populations.	□ Self-reported questionnaires—potential social desirability bias or misunderstanding; □ Intervention period not specified; □ Educational session for the control group was shorter than that of the participants receiving intervention.
Noh [70]	□ Good replicability; quasi-experimental study design; □ Conducted on female runaway youth—there is an underrepresentation of existing data/literature surrounding this population; □ Targeted problem drinking, which is what YEH or runaway adolescents turn to.	□ Homogeneity between the control and experimental groups showed no statistical difference at baseline; □ Intervention period not specified; □ Convenience sampling—lack of variety, bias in sampling; □ Small sample size.
Noh & Choi [71]	□ Culturally sensitive: addresses and emphasises the role of family that is deeply ingrained in Korean culture; □ RCT design; □ Tailored intervention specific for socio-cultural contexts; □ Uses evidence- and theory-based methods for practical applications; □ It is a comprehensive needs assessment—the program was created based on a combination of literature reviews and interviews with shelter workers and runaway adolescents residing in shelters.	□ To date, this program has not been tested for its effectiveness and efficacy and might not yield the results expected from the authors; □ Only a small selection of shelter workers and runaway adolescents were interviewed for the comprehensive needs assessment. Other extraneous factors could have been missed; □ Other variables, such as school, peers, and environmental factors, play an equally important role.
Sarmini & Sukartiningsih [67]	□ Socio-cultural specific: includes Indonesia’s strong Islamic values; □ Established by the government; □ The KAN emphasises that street children should be the responsibility of the government.	□ The KAN is only specific to Indonesia—limited generalisability; lack of replicability; □ Subjectivity and bias—relies on researcher’s interpretation; □ Small sample size; □ May not establish cause and effect due to the absence of controlled experimental conditions; □ Did not quantify any outcomes.
Solong et al. [68]	□ In-depth description of up-to-date current policies on street children.	□ Small sample size; only 2 street children were interviewed—may not be generalisable; □ Lack of replicability; time-consuming and resource-intensive; □ Selective reporting bias.

**Table 6:** Three services implemented by the Makassar City Region.

Services	Description
Community-based	□ Social services for street children were established within the community depending on the residence of the child and their family; □ The primary objective of this service was to prevent children from poor families who were at significant risk of ending up as street children. This service was a collective effort involving parents and community members [68].
Street-based	□ Social services were implemented in street environments and public spaces; □ The goal of this service was to deter street-working children from getting involved in criminal activities, with the intention of reintegrating them back with their families [68].
Centre-based	□ Dedicated institutions like orphanages were established to provide care for street children. Children were removed from the street environment and placed in these facilities; □ The primary goal of this service was to address and heal the physical, psychological, and social injuries street children experience; □ This service was provided indefinitely, allowing the children to recover from the negative impacts of street life [68].

**Table 7:** Responsibilities of the KAN.

	Responsibilities	Description
1	The government should be responsible for fulfilling their basic needs and providing support	This indicates that the government should take on a protective and caregiving role for street children, addressing their needs for safety, shelter, food, education, and other essential services [67].
2	Educational rights	Efforts for educational rights of street children are upheld; this approach indicates a commitment to addressing educational challenges faced by street children by offering various pathways for learning tailored to their individual needs and circumstances [67].
3	‘*Pak Ustadz*’—a spiritual mental advisor	A *Pak Ustadz’s* role in the KAN holds particular significance due to the country’s strong Islamic influence and values; they can offer spiritual guidance to street children, helping them connect with their faith and find a sense of purpose and identity. Additionally, as Indonesian culture is deeply rooted in Islamic traditions, *Pak Ustadz* can teach street children about cultural norms, respect, and moral values, aiding their integration into society [67].
4	Mental and behavioural guidance	This is achieved through coaching activities. It aims to provide support and guidance to street children by helping them transform their thought patterns and behaviours to align with positive societal norms. This approach addresses the unique challenges faced by street children by guiding them towards more constructive choices and behaviours that lead to better mental and behavioural outcomes.
5	Life skills-based education	These are in the form of entrepreneurial activities within the children’s village, as well as partnerships with different organisations for apprenticeship programs—helpful in acquiring valuable experience that can enhance their prospects for future employment and self-sufficiency [67].
6	Achievement culture	The KAN is actively involved in nurturing the interests and talents of street children: they have received commendable accomplishments, such as becoming champions in bicycle racing and winning vlog competitions [67]. This signifies that KAN provides a supportive environment where street children can explore their passions and talents, gain recognition for their abilities, and develop a sense of accomplishment and self-esteem [67].

## Supplementary Materials

The following supporting information can be downloaded at https://www.mdpi.com/article/10.3390/children11070864/s1. Table S1: Preferred Reporting Items for Systematic reviews and Meta-Analyses extension for Scoping Reviews (PRISMA-ScR) checklist; Table S2: Search terms used; Supplementary Materials S3: The Critical Appraisal Skills Programme (CASP) checklists.
