# Supplementary material for: Exploring Mental Health Services for Youth Experiencing Homelessness in East Asian Pacific Regions: A Systematic Scoping Review
Source: Children (Basel). 2024 Jul 17;11(7):864. doi: 10.3390/children11070864 (PMC11275148; doi:10.3390/children11070864)
Supplement: Supplementary file 1 [file children-11-00864-s001.zip › children-3038594-supplementary.pdf]

Table S1

Preferred Reporting Items for Systematic reviews and Meta-Analyses extension for Scoping Reviews (PRISMA-ScR) Checklist

SECTION	ITEM	PRISMA-ScR CHECKLIST ITEM	REPORTED ON PAGE #
**TITLE**			
Title	1	Identify the report as a scoping review.	1
**ABSTRACT**			
Structured summary	2	Provide a structured summary that includes (as applicable): background, objectives, eligibility criteria, sources of evidence, charting methods, results, and conclusions that relate to the review questions and objectives.	2
**INTRODUCTION**			
Rationale	3	Describe the rationale for the review in the context of what is already known. Explain why the review questions/objectives lend themselves to a scoping review approach.	4–5
Objectives	4	Provide an explicit statement of the questions and objectives being addressed with reference to their key elements (e.g., population or participants, concepts, and context) or other relevant key elements used to conceptualize the review questions and/or objectives.	5
**METHODS**			
Protocol and registration	5	Indicate whether a review protocol exists; state if and where it can be accessed (e.g., a Web address); and if available, provide registration information, including the registration number.	5
Eligibility criteria	6	Specify characteristics of the sources of evidence used as eligibility criteria (e.g., years considered, language, and publication status), and provide a rationale.	5
Information sources*	7	Describe all information sources in the search (e.g., databases with dates of coverage and contact with authors to identify additional sources), as well as the date the most recent search was executed.	5–6
Search	8	Present the full electronic search strategy for at least 1 database, including any limits used, such that it could be repeated.	6
Selection of sources of evidence†	9	State the process for selecting sources of evidence (i.e., screening and eligibility) included in the scoping review.	6
Data charting process‡	10	Describe the methods of charting data from the included sources of evidence (e.g., calibrated forms or forms that have been tested by the team before their use, and whether data charting was done independently or in duplicate) and any processes for obtaining and confirming data from investigators.	6
Data items	11	List and define all variables for which data were sought and any assumptions and simplifications made.	8
Critical appraisal of individual sources of evidence§	12	If done, provide a rationale for conducting a critical appraisal of included sources of evidence; describe the methods used and how this information was used in any data synthesis (if appropriate).	6
Synthesis of results	13	Describe the methods of handling and summarizing the data that were charted.	7
**RESULTS**			
Selection of sources of evidence	14	Give numbers of sources of evidence screened, assessed for eligibility, and included in the review, with reasons for exclusions at each stage, ideally using a flow diagram.	7–8 (**Table 3**)
Characteristics of sources of evidence	15	For each source of evidence, present characteristics for which data were charted and provide the citations.	8 (**Table 4**)
Critical appraisal within sources of evidence	16	If done, present data on critical appraisal of included sources of evidence (see item 12).	6–11 (**Appendix C**)

SECTION	ITEM	PRISMA-ScR CHECKLIST ITEM	REPORTED ON PAGE #
Results of individual sources of evidence	17	For each included source of evidence, present the relevant data that were charted that relate to the review questions and objectives.	8 (**Table 4**)
Synthesis of results	18	Summarize and/or present the charting results as they relate to the review questions and objectives.	8–12
**DISCUSSION**			
Summary of evidence	19	Summarize the main results (including an overview of concepts, themes, and types of evidence available), link to the review questions and objectives, and consider the relevance to key groups.	12–13
Limitations	20	Discuss the limitations of the scoping review process.	17
Conclusions	21	Provide a general interpretation of the results with respect to the review questions and objectives, as well as potential implications and/or next steps.	18–19
**FUNDING**			
Funding	22	Describe sources of funding for the included sources of evidence, as well as sources of funding for the scoping review. Describe the role of the funders of the scoping review.	20

JBI = Joanna Briggs Institute; PRISMA-ScR = Preferred Reporting Items for Systematic reviews and Meta-Analyses extension for Scoping Reviews.

* Where *sources of evidence* (see second footnote) are compiled from, such as bibliographic databases, social media platforms, and Web sites.

† A more inclusive/heterogeneous term used to account for the different types of evidence or data sources (e.g., quantitative and/or qualitative research, expert opinion, and policy documents) that may be eligible in a scoping review as opposed to only studies. This is not to be confused with *information sources* (see first footnote).

‡ The frameworks by Arksey and O'Malley (6) and Levac and colleagues (7) and the JBI guidance (4, 5) refer to the process of data extraction in a scoping review as data charting.

§ The process of systematically examining research evidence to assess its validity, results, and relevance before using it to inform a decision. This term is used for items 12 and 19 instead of "risk of bias" (which is more applicable to systematic reviews of interventions) to include and acknowledge the various sources of evidence that may be used in a scoping review (e.g., quantitative and/or qualitative research, expert opinion, and policy document).

From: Tricco AC, Lillie E, Zarin W, O'Brien KK, Colquhoun H, Levac D, et al. PRISMA Extension for Scoping Reviews (PRISMAScR): Checklist and Explanation. Ann Intern Med. 2018;169:467–473.

Table S2

Search terms used

Medline

Category	Search terms
YEH	((homeless* and (child* or youth* or adolescen* or teen* or young person* or young people*)) or street child* or street sleep* or "homeless* youth" or ill-housed person* or rough sleeper* or railway boy* or street dweller* or refugee*)<br>(Homeless persons or Homelessness or Homeless family or Homeless Shelters or Homeless Youth or Homeless single person or "outreach to the homeless" or homeless mentally ill or homeless shelter resident or Homeless Health Concerns)<br>(Runaways or Runaway children or Street Youth)
**AND**	
Mental health intervention	(mental health service* or therapeutic support* or counselling or counseling or housing program* or temporary shelter* or homeless shelter* or psychological counseling or psychological counselling or short-term temporary care or short-term care or youth homeless* shelter or non government* organisation* or non government* organization* or non-government* organisation* or non-government* organization* or NGO* or mental health care or mental health support* or cognitive behavioural therap* or cognitive behavioral therap* or CBT* or substance abuse therap* or outreach program* or outreach support* or mental health intervention* or mental health* or life counseling or life counselling or overcrowded or refugee* or emergency accommodation* or homeless* facilit* or rehabilitation* or prevention approach* or social work* or therap*)<br>Mental Health Services/ or Child Guidance/ or Community Mental Health Services/ or Counseling/ or Emergency Services, Psychiatric/ or Social Work, Psychiatric/<br>(health service, mental or health services, mental or hygiene service, mental or hygiene services, mental or mental health service or mental health services or mental hygiene service or mental hygiene services or service, mental health or service, mental hygiene or services, mental health or services, mental hygiene)
**AND**	
EAP countries	(east asia* pacific or east asia* pacific countr* or cambodia* or china or chinese* or hong kong or indonesia* or japan* or south korea* or lao* pdr or macau or macanese or malaysia* or mongolia* or myanmar or pacific island* or papua new guinea or papuans or philippin* or filipin* or the philippine* or singapore* or taiwan* or thai or timor-leste or vietnam*)<br>exp Cambodia/ or exp Indochina/ or exp Indonesia/ or exp Laos/ or exp Malaysia/ or exp Myanmar/ or exp Philippines/ or exp Singapore/ or exp Thailand/ or exp Timor-Leste/ or exp Vietnam/ or exp China/ or exp Japan/ or exp Korea/ or exp Mongolia/ or exp Taiwan/ or exp Indonesia/ or exp Japan/ or exp Macau/ or exp Philippines/ or exp Taiwan/
**Results: 120**	

PsychInfo

Category	Search terms
YEH	((homeless* and (child* or youth* or adolescen* or teen*)) or street child* or street sleep* or ill-housed person* or rough sleep* or street dwell* or railway boy*)<br>(child, homeless or child, street or children, homeless or children, street or homeless child or homeless children or homeless youth or homeless youths or runaway or runaways or street child or street children or street youth or youth, homeless or youth, street or youths, homeless or youths, street)
**AND**	

Mental health intervention	(mental health service* or therapeutic support* or counselling or counseling or housing program* or temporary shelter* or homeless shelter* or psychological counseling or psychological counselling or short-term temporary care or short-term care or youth homeless* shelter or non government* organisation* or non government* organization* or non-government* organisation* or non-government* organization* or NGO* or mental health care or mental health support* or cognitive behavioural therap* or cognitive behavioral therap* or CBT* or substance abuse therap* or outreach program* or outreach support* or mental health intervention* or mental health* or life counseling or life counselling or overcrowded or centre base* or center base* or emergency accommodation* or homeless* facilit* or food bank* or rehabilitation* or prevention approach* or social work* or therap*)<br>(Rehabilitation Counseling or School Counseling or Aftercare or School Counseling or Mental Health Services or Community Mental Health Services or Early Intervention or Family Intervention or School Based Intervention or Mental Health Programs or Crisis Intervention Services or Hot Line Services or Suicide Prevention Centers or Home Visiting Programs or Suicide Prevention Centers)
**AND**	
EAP countries	(east asia* pacific or east asia* pacific countr* or cambodia* or china or chinese* or hong kong or indonesia* or japan* or south korea* or lao* pdr or macau or macanese or malaysia* or mongolia* or myanmar or pacific island* or papua new guinea or papuans or philippin* or filipin* or the philippine* or singapore* or taiwan* or thai or timor-leste or vietnam*)<br>(Pacific Islanders or Asia Southeastern or Asia Eastern)
**Results: 153**	

PubMed

Category	Search terms
YEH	(((((((((((((homeless*)) AND (child*)) OR (youth*)) OR (adolescen*)) OR (teen*)) OR (ill-housed person*)) OR ("homeless teen")) OR ("street sleeper"[tiab:~0])) OR ("children of the street"[tiab:~0])) OR ("street youth")) OR ("runaway* child*")) OR ("runaway* adolescen*")) OR ("street child*")
**AND**	
Mental health intervention	((((((((((((((((((((((((((((((((((mental health service*) OR (therapeutic support*)) OR (counselling)) OR (counseling)) OR ("housing program*")) OR ("temporary shelter*")) OR ("homeless shelter*")) OR ("psychological counseling")) OR ("psychological counselling")) OR ("temporary care")) OR ("short-term care")) OR ("youth homeless* shelter"[tiab:~10])) OR ("non government* organisation")) OR ("non government* organization")) OR ("non-government* organisation")) OR ("non-government* organization")) OR ("NGO"[tiab])) OR ("mental health care")) OR ("mental health support*")) OR ("cognitive behavioural therap*")) OR ("cognitive behavioral therap*")) OR (CBT[tiab])) OR ("substance abuse therap*")) OR ("outreach program*")) OR ("outreach support*")) OR ("mental health intervention")) OR ("rehabilitation counseling")) OR ("community mental health service*")) OR ("family intervention*")) OR ("mental health program*")) OR ("crisis intervention service*")) OR ("hot line service*")) OR ("suicide prevention center*")) OR (psychotherap*)) OR ("homeless intervention*")
**AND**	
EAP countries	((((((((((((((((((((((((((((east asia* pacific) OR (east asia* pacific countr*)) OR (cambodia*)) OR (china)) OR (chinese)) OR (hong kong)) OR (indonesia*)) OR (japan*)) OR (south korea*)) OR (lao* pdr)) OR (macau)) OR (macanese)) OR (malaysia*)) OR (mongolia*)) OR (myanmar)) OR (pacific island*)) OR (papua new guinea)) OR (papuans)) OR (philippin*)) OR ("the philippin*")) OR (filipin*)) OR (singapore*)) OR (taiwan*)) OR

	(thai)) OR (timor-leste)) OR (vietnam*)) OR ("south-east asia*")) OR ("southeast asia*")) OR ("east asia*")

Results: 2,546

Scopus

Category	Search terms
YEH	"homeless*" OR "homeless* youth" OR "homeless* child*" OR "homeless* adolescen*" OR "homeless* teen" OR " homeless* young person*" OR "homeless* young people*" OR "street child*" OR "street sleeper" OR "homeless* youth" OR "ill-housed person" OR "street youth" OR runaway* OR "runaway youth*" OR "street youth*" OR "rough sleep*" OR "railway boy*" OR "street dwell*" OR "refugee*"
**AND**	
Mental health intervention	"mental health service*" OR "mental health intervention*" OR "psychological intervention*" OR "therap* support*" OR counseling OR "counselling" OR "psychological counseling" OR "psychological counselling" OR "short-term temporary care" OR "short-term care" OR "youth homeless* shelter" OR "homeless* shelter*" OR "non-government* organisation*" OR "non-governmen* organization" OR NGO* OR "mental health care" OR "mental health support" OR "cognitive behavioural therap*" OR "cognitive behavioral therap*" OR CBT OR "outreach program*" OR "outreach support*" OR "outreach work*" OR "homeless* policy*" OR "homeless* policies" OR "homeless* law*" OR "policies" or "policy" OR "community service*" OR "community mental health service*" OR "emergency service*" OR "family therap*" OR "family intervention*" OR "mental health program*" OR "crisis intervention* service*" OR "hotline service*" OR "school based intervention*" OR "suicide prevention cent*" OR "home visiting program*" OR "community program*" OR "family based intervention*" OR "family based intervention*" OR "emergency accommodation*" OR "homeless* facilit*" OR "food bank*" OR rehabilitation* OR "social work*"
**AND**	
EAP countries	"east asia* pacific" OR "east asia* pacific countr*" OR cambodia* OR china OR chinese OR "hong kong" OR indonesia* OR japan* or "south korea*" OR "lao* pdr" OR macau OR macanese OR malaysia* OR mongolia* OR myanmar OR "pacific island*" OR "papua new guinea" OR papuans OR philippin* OR "the philippin*" OR filipin* OR singapore* OR taiwan* OR thai* OR "timor-leste" OR viet*

Results: 613

Web of Science

Category	Search terms
YEH	((((((((((((((ALL=(homeless*)) AND ALL=(youth*)) OR ALL=(child*)) OR ALL=(adolescen*)) OR ALL=(teen)) OR ALL=("street child*")) OR ALL=("street sleeper")) OR ALL=("ill-housed person*")) OR ALL=("street youth*")) OR ALL=(runaway*)) OR ALL=("runaway youth*")) OR ALL=("homeless youth*")) OR ALL=("rough sleep*")) OR ALL=("railway boy*")) OR ALL=("street dwell*")) OR ALL=("refugee*")) OR ALL=("left behind child*")
**AND**	
Mental health intervention	((((((((((((((((((((((((((((((((((((ALL=("mental health service*")) OR ALL=("mental health intervention*")) OR ALL=("psychological intervention*")) OR ALL=("therap* support*")) OR ALL=(counseling)) OR ALL=(counselling)) OR ALL=("psychological counseling")) OR ALL=("psychological counselling")) OR ALL=("short-term temporary care")) OR ALL=("short-term care")) OR ALL=("youth homeless* shelter*")) OR ALL=("homeless* shelter")) OR ALL=("non-government* organisation*")) OR ALL=("non-government* organization*")) OR ALL=(NGO*)) OR ALL=("mental health care")) OR ALL=("mental health support*")) OR ALL=("cognitive behavioural therap*")) OR ALL=("cognitive behavioral therap*")) OR ALL=(CBT)) OR ALL=("motivation* interview*")) OR

	ALL=("substance abuse therap*")) OR ALL=("outreach program*")) OR ALL=("outreach support*")) OR ALL=("outreach work*")) OR ALL=(policy)) OR ALL=("mental health*")) OR ALL=("life counseling")) OR ALL=("life counselling")) OR ALL=("overcrowded")) OR ALL=("centre based")) OR ALL=("center based")) OR ALL=("emergency accommodation")) OR ALL=("homeless* facilit*")) OR ALL=("food bank*")) OR ALL=("rehabilitation*")) OR ALL=("social work*")) OR ALL=("therap*")
**AND**	
EAP countries	((((((((((((((((((((((((ALL=("east asia* pacific")) OR ALL=("east asia* pacific countr*")) OR ALL=(cambodia*)) OR ALL=(china)) OR ALL=(chinese)) OR ALL=("hong kong")) OR ALL=(indonesia*)) OR ALL=(japan*)) OR ALL=("south korea*")) OR ALL=("lao* pdr")) OR ALL=(macau)) OR ALL=(manganese)) OR ALL=(malaysia*)) OR ALL=(mongolia*)) OR ALL=(myanmar)) OR ALL=("pacific island*")) OR ALL=("papua new guinea")) OR ALL=(papuans)) OR ALL=(philippin*)) OR ALL=("the philippin*")) OR ALL=("filipin*")) OR ALL=(singapore*)) OR ALL=(tawain*)) OR ALL=(thai*)) OR ALL=("timor-leste")) OR ALL=(vietnam*)
**Results: 92**	

Supplementary material 3

The Critical Appraisal Skills Programme (CASP) checklists

www.casp-uk.net
info@casp-uk.net
Summertown Pavilion, Middle Way Oxford OX2 7LG

CASP Checklist: 10 questions to help you make sense of a Qualitative research

How to use this appraisal tool: Three broad issues need to be considered when appraising a qualitative study:

- Are the results of the study valid? (Section A)
- What are the results? (Section B)
- Will the results help locally? (Section C)

The 10 questions on the following pages are designed to help you think about these issues systematically. The first two questions are screening questions and can be answered quickly. If the answer to both is "yes", it is worth proceeding with the remaining questions. There is some degree of overlap between the questions, you are asked to record a "yes", "no" or "can't tell" to most of the questions. A number of italicised prompts are given after each question. These are designed to remind you why the question is important. Record your reasons for your answers in the spaces provided.

About: These checklists were designed to be used as educational pedagogic tools, as part of a workshop setting, therefore we do not suggest a scoring system. The core CASP checklists (randomised controlled trial & systematic review) were based on JAMA 'Users' guides to the medical literature 1994 (adapted from Guyatt GH, Sackett DL, and Cook DJ), and piloted with health care practitioners.

For each new checklist, a group of experts were assembled to develop and pilot the checklist and the workshop format with which it would be used. Over the years overall adjustments have been made to the format, but a recent survey of checklist users reiterated that the basic format continues to be useful and appropriate.

Referencing: we recommend using the Harvard style citation, i.e.: *Critical Appraisal Skills Programme (2018). CASP (insert name of checklist i.e. Qualitative) Checklist. [online] Available at: URL. Accessed: Date Accessed.*

©CASP this work is licensed under the Creative Commons Attribution – Non-Commercial-Share A like. To view a copy of this license, visit http://creativecommons.org/licenses/by-nc-sa/3.0/ www.casp-uk.net

CASP
Critical Appraisal
Skills Programme

Paper for appraisal and reference: Brilliantes-Evangelista (2013) An evaluation of visual arts and poetry as therapeutic interventions with abused adolescents

Section A: Are the results valid?

1. Was there a clear statement of the aims of the research?

Yes ✔
Can't Tell ☐
No ☐

HINT: Consider
- what was the goal of the research
- why it was thought important
- its relevance

Comments:
Clear statement of aims, rationale and relevancy.
The goal of the research was to explore the viability of visual arts and poetry as effective interventions for abused adolescents. Child abuse is one of the growing social concerns in the Philippines and there are a number of psychological problems among children and adolescents who have been physically and sexually abused. Art is interwoven in the Filipino culture, yet there are no published local models for the use of arts specific to psychotherapy.

2. Is a qualitative methodology appropriate?

Yes ✔
Can't Tell ☐
No ☐

HINT: Consider
- If the research seeks to interpret or illuminate the actions and/or subjective experiences of research participants
- Is qualitative research the right methodology for addressing the research goal

Comments:
Visual arts and poetry intervention sessions explored subjective experiences of the participants by using various means of media, art and modality to illuminate themes such as fears, personal life story, unwanted characteristics, view of the self and of family members. Researcher examined the experiences of participants by looking at their behaviours, artwork and interview responses over the course of the entire sessions. According to the researcher, these were content analysed.

Is it worth continuing?

3. Was the research design appropriate to address the aims of the research?

Yes ✔
Can't Tell ☐
No ☐

HINT: Consider
- if the researcher has justified the research design (e.g. have they discussed how they decided which method to use)

Comments:
Research design appropriate as researcher used quasi-experimental (repeated measures design) for psychopathology symptomatoology pre-test, mid-assessment and post-test and qualitative methodologies for subjective experiences.

CASP Critical Appraisal Skills Programme

4. Was the recruitment strategy appropriate to the aims of the research?

Yes ✔
Can't Tell ☐
No ☐

HINT: Consider
- If the researcher has explained how the participants were selected
- If they explained why the participants they selected were the most appropriate to provide access to the type of knowledge sought by the study
- If there are any discussions around recruitment (e.g. why some people chose not to take part)

Comments: Researcher selected participants from shelters around Metro Manila based on the depression and PTSD measures. Researcher did not explain why they chose shelters. However, it seems most appropriate due to its accessibility and population; researcher explained that the participants were physically and sexually abused. Researcher discussed that before the post-test was conducted, four from the control group dropped out, three ran away and one went back to his family.
Control group was a no-treatment group but were given pre- and post-test of the depression and PTSD scales.

5. Was the data collected in a way that addressed the research issue?

Yes ✔
Can't Tell ☐
No ☐

HINT: Consider
- If the setting for the data collection was justified
- If it is clear how data were collected (e.g. focus group, semi-structured interview etc.)
- If the researcher has justified the methods chosen
- If the researcher has made the methods explicit (e.g. for interview method, is there an indication of how interviews are conducted, or did they use a topic guide)
- If methods were modified during the study. If so, has the researcher explained how and why
- If the form of data is clear (e.g. tape recordings, video material, notes etc.)
- If the researcher has discussed saturation of data

Comments: Clear data collection and comprehensive information regarding instruments used. Instruments used (e.g., depression and PTSD rating scales) were translated in Filipino and back translated to ensure consistency of the Filipino version to the original scale. Researcher explained that one item was deleted during pre-test because of a misinterpretation from a test taker.

6. Has the relationship between researcher and participants been adequately considered?

Yes ☐
Can't Tell ☐
No ☑

HINT: Consider
- If the researcher critically examined their own role, potential bias and influence during (a) formulation of the research questions (b) data collection, including sample recruitment and choice of location
- How the researcher responded to events during the study and whether they considered the implications of any changes in the research design

Comments:
Researcher did not critically examine their own role, potential bias and influence during the formulation of research questions and data collection. Researcher did not indicate or mention any events that may have happened during the study.

Section B: What are the results?

7. Have ethical issues been taken into consideration?

Yes ☐
Can't Tell ☐
No ☑

HINT: Consider
- If there are sufficient details of how the research was explained to participants for the reader to assess whether ethical standards were maintained
- If the researcher has discussed issues raised by the study (e.g. issues around informed consent or confidentiality or how they have handled the effects of the study on the participants during and after the study)
- If approval has been sought from the ethics committee

Comments:
Author did not mention whether approval was sought from the ethics committee. Author did not discuss informed consent or confidentiality in the paper, however, participant names were not included.

8. Was the data analysis sufficiently rigorous?

Yes ☑ Can't Tell ☐ No ☐

HINT: Consider
- If there is an in-depth description of the analysis process
- If thematic analysis is used. If so, is it clear how the categories/themes were derived from the data
- Whether the researcher explains how the data presented were selected from the original sample to demonstrate the analysis process
- If sufficient data are presented to support the findings
- To what extent contradictory data are taken into account
- Whether the researcher critically examined their own role, potential bias and influence during analysis and selection of data for presentation

Comments:
Comprehensive and in-depth description of the analysis process. Clear tables used for PTSD and depression scores for control group, visual arts group and poetry group, pre-test, mid-test and post-test.

Data from poetry and visual arts sessions were content analysed. However, they were not analysed by an art or poetry therapist but by a researcher (a clinical psychologist).

9. Is there a clear statement of findings?

Yes ☑ Can't Tell ☐ No ☐

HINT: Consider whether
- If the findings are explicit
- If there is adequate discussion of the evidence both for and against the researcher's arguments
- If the researcher has discussed the credibility of their findings (e.g. triangulation, respondent validation, more than one analyst)
- If the findings are discussed in relation to the original research question

Comments:

Clear statement of findings that support the original research question. Adequate discussion of the evidence both for and against with reference to existing literature. Researcher lists study limitations and recommendations for future researches.

CASP
Critical Appraisal Skills Programme

Section C: Will the results help locally?

10. How valuable is the research?

HINT: Consider
- If the researcher discusses the contribution the study makes to existing knowledge or understanding (e.g. do they consider the findings in relation to current practice or policy, or relevant research-based literature
- If they identify new areas where research is necessary
- If the researchers have discussed whether or how the findings can be transferred to other populations or considered other ways the research may be used

Comments: Results from visual arts and poetry intervention demonstrates an implication of how we should view psychotherapy. Although alleviating depression and PTSD symptoms may not be guaranteed, these interventions seemed to have been helpful in other ways such as empowering the abused adolescent in having a meaningful existence. Author suggested that psychological healing may not just be removing symptoms of psychopathology, but psychotherapy must be keener in helping individuals have a more meaningful existence and quality of life.
Art and poetry therapy is not institutionalised in the Philippines, thus, visual arts group was not designed by a certified art therapist, and the poetry facilitator was also not a certified poetry therapist. Furthermore, each group only had eight intervention sessions and the author concluded that perhaps more sessions were needed to reduce depression and PTSD symptomatology. This should be considered for future research.

CASP Randomised Controlled Trial Standard Checklist:
11 questions to help you make sense of a randomised controlled trial (RCT)

Main issues for consideration: Several aspects need to be considered when appraising a randomised controlled trial:

- Is the basic study design valid for a randomised controlled trial? (Section A)
- Was the study methodologically sound? (Section B)
- What are the results? (Section C)
- Will the results help locally? (Section D)

The 11 questions in the checklist are designed to help you think about these aspects systematically.

How to use this appraisal tool: The first three questions (Section A) are screening questions about the validity of the basic study design and can be answered quickly. If, in light of your responses to Section A, you think the study design is valid, continue to Section B to assess whether the study was methodologically sound and if it is worth continuing with the appraisal by answering the remaining questions in Sections C and D.

Record 'Yes', 'No' or 'Can't tell' in response to the questions. Prompts below all but one of the questions highlight the issues it is important to consider. Record the reasons for your answers in the space provided. As CASP checklists were designed to be used as educational/teaching tools in a workshop setting, we do not recommend using a scoring system.

About CASP Checklists: The CASP RCT checklist was originally based on JAMA Users' guides to the medical literature 1994 (adapted from Guyatt GH, Sackett DL and Cook DJ), and piloted with healthcare practitioners. This version has been updated taking into account the CONSORT 2010 guideline (http://www.consort-statement.org/consort-2010, accessed 16 September 2020).

Citation: CASP recommends using the Harvard style, i.e., *Critical Appraisal Skills Programme (2021). CASP (insert name of checklist i.e. Randomised Controlled Trial) Checklist. [online] Available at: insert URL. Accessed: insert date accessed.*

©CASP this work is licensed under the Creative Commons Attribution – Non-Commercial- Share A like. To view a copy of this licence, visit https://creativecommons.org/licenses/by-sa/4.0/

CASP
Critical Appraisal
Skills Programme

Study and citation: Hyun, Chung & Lee (2005) The effect of cognitive-behavioural group therapy on the self-esteem, depression and self-efficacy of runaway adolescents in a shelter in South Korea

Section A: Is the basic study design valid for a randomised controlled trial?

**1. Did the study address a clearly focused research question?**<br>*CONSIDER:*<br>☐ *Was the study designed to assess the outcomes of an intervention?*<br>☐ *Is the research question 'focused' in terms of:*<br>• *Population studied*<br>• *Intervention given*<br>• *Comparator chosen*<br>• *Outcomes measured?*	Yes ☐ No ☐ Can't tell ☐<br>Study was designed to assess the effectiveness of cognitive-behavioural group therapy for self-esteem, depression and self-efficacy among runaway adolescents.<br><br>Research question focused:<br>Population: Male runaway adolescents residing in a shelter<br>Intervention: Cognitive-behavioural group therapy<br>Comparator: Control group pre-test and post-test design<br>Outcomes measured: Self-esteem, depression and self-efficacy
**2. Was the assignment of participants to interventions randomised?**<br>*CONSIDER:*<br>• *How was randomisation carried out? Was the method appropriate?*<br>• *Was randomisation sufficient to eliminate systematic bias?*<br>• *Was the allocation sequence concealed from investigators and participants?*	Yes ☐ No ☐ Can't tell ☐<br>Research participants were randomly assigned to the experimental group or the control group.
**3. Were all participants who entered the study accounted for at its conclusion?**<br>*CONSIDER:*<br>• *Were losses to follow-up and exclusions after randomisation accounted for?*<br>• *Were participants analysed in the study groups to which they were randomised (intention-to-treat analysis)?*<br>• *Was the study stopped early? If so, what was the reason?*	Yes ☐ No ☐ Can't tell ☐<br>Researcher documented that there were 32 adolescents enrolled in the study, however, five were excluded from the analyses (two in the experimental; three in the experimental) because they left the facility before the post-test.

Section B: Was the study methodologically sound?

	Yes	No	Can't tell
**4.**			
☐ **Were the participants 'blind' to intervention they were given?**	☐	☐	☐
☐ **Were the investigators 'blind' to the intervention they were giving to participants?**	☐	☐	☐
☐ **Were the people assessing/analysing outcome/s 'blinded'?**	☐	☐	☐

**5. Were the study groups similar at the start of the randomised controlled trial?**<br>*CONSIDER:*<br>☐ *Were the baseline characteristics of each study group (e.g. age, sex, socio-economic group) clearly set out?*<br>☐ *Were there any differences between the study groups that could affect the outcome/s?*	Yes ☐ No ☐ Can't tell ☐<br>The researcher explained that the study was conducted in one shelter in South Korea to maintain homogeneity between the experimental and control group.<br><br>Participants were all male. Baseline characteristics of both control and experimental group in terms of demographic characteristics and pre-test values were set out and compared. Researcher documented no significant differences in self-esteem, depression and self-esteem and baseline characteristics between the experimental and control group.

CASP
Critical Appraisal
Skills Programme

**6.**	**Apart from the experimental intervention, did each study group receive the same level of care (that is, were they treated equally)?**<br><br>*CONSIDER:*<br>☐ *Was there a clearly defined study protocol?*<br>☐ *If any additional interventions were given (e.g. tests or treatments), were they similar between the study groups?*<br>☐ *Were the follow-up intervals the same for each study group?*	Yes ☐ No ☐ Can't tell ☐<br><br>Authors noted that all participants were assured they could withdraw from the study at any time and that this would have no effect on their relationship with the staff or on the treatment that they received in the shelter.<br><br>Subjects in both experimental and control group were assessed for self-esteem, depression and self-efficacy within the same time frames pre-test and post-test.<br><br>New residents at the shelter were able to participate in the program, and were provided with supplementary sessions to make up for the parts of the CBT program that they had missed.

Section C: What are the results?

**7.**	**Were the effects of intervention reported comprehensively?**<br><br>*CONSIDER:*<br>• *Was a power calculation undertaken?*<br>• *What outcomes were measured, and were they clearly specified?*<br>• *How were the results expressed? For binary outcomes, were relative and absolute effects reported?*<br>• *Were the results reported for each outcome in each study group at each follow-up interval?*<br>• *Was there any missing or incomplete data?*<br>• *Was there differential drop-out between the study groups that could affect the results?*<br>• *Were potential sources of bias identified?*<br>• *Which statistical tests were used?*<br>• *Were p values reported?*	Yes ☐ No ☐ Can't tell ☐<br><br>Effects of intervention and baseline comparisons both pre-test and post-test were reported comprehensively using clear tables.<br><br>Homogeneity between the experimental group and the control group in terms of demographics and pre-test values (self-esteem, depression, self-efficacy) were tested using the Fisher's Exact probability and the Mann-Whitney U test. For the effects of CBT on self-esteem, depression and self-efficacy, the Wilcoxon signed rank test was used. P values were reported as well as standard deviations.<br><br>No data was incomplete or missing. Participants who enrolled but subsequently dropped out of the study before post-test were excluded from analyses. Therefore, these would not have affected the results.
**8.**	**Was the precision of the estimate of the intervention or treatment effect reported?**<br><br>*CONSIDER:*<br>• *Were confidence intervals (CIs) reported?*	Yes ☐ No ☐ Can't tell ☐<br><br>CIs were not reported.
**9.**	**Do the benefits of the experimental intervention outweigh the harms and costs?**<br><br>*CONSIDER:*<br>☐ *What was the size of the intervention or treatment effect?*<br>☐ *Were harms or unintended effects reported for each study group?*<br>☐ *Was a cost-effectiveness analysis undertaken? (Cost-effectiveness analysis allows a comparison to be made between different interventions used in the care of the same condition or problem.)*	Yes ☐ No ☐ Can't tell ☐<br><br>Intervention group received eight weekly sessions of the treatment program.<br><br>Small number of subjects were selected. Sample size in this study was not large enough to permit the assumption of normality on the study variables. However, the nonparametric test was used.<br><br>Harms or unintended effects were not reported for each study group; a cost-effectiveness analysis was not undertaken.

CASP
Critical Appraisal
Skills Programme

Section D: Will the results help locally?

**10. Can the results be applied to your local population/in your context?**<br><br>*CONSIDER:*<br>• *Are the study participants similar to the people in your care?*<br>• *Would any differences between your population and the study participants alter the outcomes reported in the study?*<br>• *Are the outcomes important to your population?*<br>• *Are there any outcomes you would have wanted information on that have not been studied or reported?*<br>• *Are there any limitations of the study that would affect your decision?*	Yes ☐ No ☐ Can't tell ☐<br><br>Populations in Hyun, Chun and Lee's (2005) study and my systematic review were both youth experiencing homelessness. The authors only recruited male participants. This is a limitation, however, the results from this study are beneficial for the systematic review's findings on different mental health services for this population. It would be interesting to see if there were differences in male and female treatment responses.
**11. Would the experimental intervention provide greater value to the people in your care than any of the existing interventions?**<br><br>*CONSIDER:*<br>☐ *What resources are needed to introduce this intervention taking into account time, finances, and skills development or training needs?*<br>☐ *Are you able to disinvest resources in one or more existing interventions in order to be able to re-invest in the new intervention?*	Yes ☐ No ☐ Can't tell ☐<br><br>Recruiting youth experiencing homelessness in the West would be particularly difficult, and would probably require looking into shelters and/or care homes just as the authors did. It would require multidisciplinary collaboration and stakeholders for CBT interventions.

APPRAISAL SUMMARY: *Record key points from your critical appraisal in this box. What is your conclusion about the paper? Would you use it to change your practice or to recommend changes to care/interventions used by your organisation? Could you judiciously implement this intervention without delay?*

This is a comprehensive paper that clearly states the psychological issues of youth homelessness or runaway adolescents, why intervention is important for this population and the effects of CBT on self-esteem, depression and self-efficacy. This study demonstrated that CBT is an effective intervention for youth experiencing homelessness, as it decreased depression symptomatology and increased self-efficacy among participants.

The CBT developed in the study is deemed suitable for application to similar populations within the East Asia Pacific regions, suggesting potential benefits for addressing psychological issues among youth in this area. However, implementing this intervention in Western contexts may pose challenges due to cultural differences, indicating potential barriers to its effectiveness or acceptance in Western settings.

Female participants should be conducted in future research.

Critical Appraisal Skills Programme

www.casp-uk.net
info@casp-uk.net
Summertown Pavilion, Middle Way Oxford OX2 7LG

CASP Checklist: 11 questions to help you make sense of a **Case Control Study**

How to use this appraisal tool: Three broad issues need to be considered when appraising a case control study:

- Are the results of the study valid? (Section A)
- What are the results? (Section B)
- Will the results help locally? (Section C)

The 11 questions on the following pages are designed to help you think about these issues systematically. The first three questions are screening questions and can be answered quickly. If the answer to both is "yes", it is worth proceeding with the remaining questions. There is some degree of overlap between the questions, you are asked to record a "yes", "no" or "can't tell" to most of the questions. A number of italicised prompts are given after each question. These are designed to remind you why the question is important. Record your reasons for your answers in the spaces provided.

About: These checklists were designed to be used as educational pedagogic tools, as part of a workshop setting, therefore we do not suggest a scoring system. The core CASP checklists (randomised controlled trial & systematic review) were based on JAMA 'Users' guides to the medical literature 1994 (adapted from Guyatt GH, Sackett DL, and Cook DJ), and piloted with health care practitioners.

For each new checklist, a group of experts were assembled to develop and pilot the checklist and the workshop format with which it would be used. Over the years overall adjustments have been made to the format, but a recent survey of checklist users reiterated that the basic format continues to be useful and appropriate.

Referencing: we recommend using the Harvard style citation, i.e.: *Critical Appraisal Skills Programme (2018). CASP (insert name of checklist i.e. Case Control Study) Checklist. [online] Available at: URL. Accessed: Date Accessed.*

©CASP this work is licensed under the Creative Commons Attribution – Non-Commercial-Share A like. To view a copy of this license, visit http://creativecommons.org/licenses/by-nc-sa/3.0/ www.casp-uk.net

CASP
Critical Appraisal
Skills Programme

Paper for appraisal and reference: Miles (2000) Drawing together hope: 'listening' to militarised children

Section A: Are the results of the trial valid?

1. Did the study address a clearly focused issue?

Yes ✔
Can't Tell ☐
No ☐

HINT: An issue can be 'focused' In terms of
- the population studied
- Whether the study tried to detect a beneficial or harmful effect
- the risk factors studied

Comments:

The research population studied was focused. The focus was on orphaned refugee and militarised children and how art is used as a tool in understanding the child's view of self and hope for the future.

2. Did the authors use an appropriate method to answer their question?

Yes ✔
Can't Tell ☐
No ☐

HINT: Consider
- Is a case control study an appropriate way of answering the question under the circumstances
- Did it address the study question

Comments:

Case control is appropriate considering the nature of the participants in the study. It addresses the research question.

CASP
Critical Appraisal
Skills Programme

Is it worth continuing?

3. Were the cases recruited in an acceptable way?

Yes ✔
Can't Tell ☐
No ☐

HINT: We are looking for selection bias which might compromise validity of the findings

- are the cases defined precisely
- were the cases representative of a defined population (geographically and/or temporally)
- was there an established reliable system for selecting all the cases
- are they incident or prevalent
- is there something special about the cases
- is the time frame of the study relevant to disease/exposure
- was there a sufficient number of cases selected
- was there a power calculation

Comments:

Not a lot of cases have been conducted on orphaned militarised children which is what makes this case special. These children are a distinct, and at-risk population.

4. Were the controls selected in an acceptable way?

Yes ☐
Can't Tell ✔
No ☐

HINT: We are looking for selection bias which might compromise the generalisability of the findings

- were the controls representative of the defined population (geographically and/or temporally)
- was there something special about the controls
- was the non-response high, could non-respondents be different in any way
- are they matched, population based or randomly selected
- was there a sufficient number of controls selected

Comments:

Participants were recruited in a somewhat acceptable way considering the location. Participants were representative of a defined population (orphaned militarised children) in South East Asia in military centres.

Rather than focusing on counselling over the traumas of the past, the researcher focused on children's perception of hope for the future of themselves.

Participants were invited to participate and they all did. Sample size was 60: 52 boys and 8 girls, aged 9-16 years.

Translators were the children's teachers who were also refugees in the military centres - potential bias or mistranslation.

5. Was the exposure accurately measured to minimise bias?

Yes ☐
Can't Tell ☑
No ☐

HINT: We are looking for measurement, recall or classification bias

- was the exposure clearly defined and accurately measured
- did the authors use subjective or objective measurements
- do the measures truly reflect what they are supposed to measure (have they been validated)
- were the measurement methods similar in the cases and controls
- did the study incorporate blinding where feasible
- is the temporal relation correct (does the exposure of interest precede the outcome)

Comments:

Art was used as a tool to understand the child's subjective view of themselves and hope for the future. Children were free to draw whatever they wanted and were individually asked to explain what they had drawn.

This child-centred approach used subjective measures. However, this may not truly reflect what they are supposed to measure (how they view themselves; hope for the future) because children's vocabulary is limited according to their age and ability, thus, open to misinterpretation.

6. (a) Aside from the experimental intervention, were the groups treated equally?

HINT: List the ones you think might be important, that the author may have missed

- genetic
- environmental
- socio-economic

List:

Not applicable.

6. (b) Have the authors taken account of the potential confounding factors in the design and/or in their analysis?

Yes ☑
Can't Tell ☐
No ☐

HINT: Look for

- restriction in design, and techniques e.g. modelling, stratified-, regression-, or sensitivity analysis to correct, control or adjust for confounding factors

Comments:

The author had taken into account potential confounding factors such as art and language misinterpretation.

CASP Critical Appraisal Skills Programme

Section B: What are the results?

7. How large was the treatment effect?

HINT: Consider
- what are the bottom line results
- is the analysis appropriate to the design
- how strong is the association between exposure and outcome (look at the odds ratio)
- are the results adjusted for confounding, and might confounding still explain the association
- has adjustment made a big difference to the OR

Comments:

The author states that art was a useful tool to opening up communication between children and adults.

8. How precise was the estimate of the treatment effect?

HINT: Consider
- size of the p-value
- size of the confidence intervals
- have the authors considered all the important variables
- how was the effect of subjects refusing to participate evaluated

Comments:

Author did not consider risks associated with being orphaned and militarised children such as psychopathology. These could have affected the way the children drew hope for the future and/or view of the self.

CASP
Critical Appraisal Skills Programme

9. Do you believe the results?

Yes ✔

No ☐

HINT: Consider
- big effect is hard to ignore!
- Can it be due to chance, bias, or confounding
- are the design and methods of this study sufficiently flawed to make the results unreliable
- consider Bradford Hills criteria (e.g. time sequence, does-response gradient, strength, biological plausibility)

Comments:
Orphaned militarised children are a distinct and at-risk population. This study could have been conducted better but considering the nature of the population, location and lack of resources and funding the author had, the results illustrated the importance of art in understanding a child's mind. This could inform future research and practice; further research should be conducted on orphaned militarised children.

Section C: Will the results help locally?

10. Can the results be applied to the local population?

Yes ✔

Can't Tell ☐

No ☐

HINT: Consider whether
- the subjects covered in the study could be sufficiently different from your population to cause concern
- your local setting is likely to differ much from that of the study
- can you quantify the local benefits and harms

Comments:
The results will help inform my systematic review. However, it is important to note that these results are outdated.

11. Do the results of this study fit with other available evidence?

Yes ✔

Can't Tell ☐

No ☐

HINT: Consider
- all the available evidence from RCT's Systematic Reviews, Cohort Studies, and Case Control Studies as well, for consistency

Comments:
The author discusses the contribution the study makes to existing literature and knowledge for consistency.

Remember One observational study rarely provides sufficiently robust evidence to recommend changes to clinical practice or within health policy decision making. However, for certain questions observational studies provide the only evidence. Recommendations from observational studies are always stronger when supported by other evidence.

CASP Randomised Controlled Trial Standard Checklist:
11 questions to help you make sense of a randomised controlled trial (RCT)

Main issues for consideration: Several aspects need to be considered when appraising a randomised controlled trial:

- Is the basic study design valid for a randomised controlled trial? (Section A)
- Was the study methodologically sound? (Section B)
- What are the results? (Section C)
- Will the results help locally? (Section D)

The 11 questions in the checklist are designed to help you think about these aspects systematically.

How to use this appraisal tool: The first three questions (Section A) are screening questions about the validity of the basic study design and can be answered quickly. If, in light of your responses to Section A, you think the study design is valid, continue to Section B to assess whether the study was methodologically sound and if it is worth continuing with the appraisal by answering the remaining questions in Sections C and D.

Record 'Yes', 'No' or 'Can't tell' in response to the questions. Prompts below all but one of the questions highlight the issues it is important to consider. Record the reasons for your answers in the space provided. As CASP checklists were designed to be used as educational/teaching tools in a workshop setting, we do not recommend using a scoring system.

About CASP Checklists: The CASP RCT checklist was originally based on JAMA Users' guides to the medical literature 1994 (adapted from Guyatt GH, Sackett DL and Cook DJ), and piloted with healthcare practitioners. This version has been updated taking into account the CONSORT 2010 guideline (http://www.consort-statement.org/consort-2010, accessed 16 September 2020).

Citation: CASP recommends using the Harvard style, i.e., *Critical Appraisal Skills Programme (2021). CASP (insert name of checklist i.e. Randomised Controlled Trial) Checklist. [online] Available at: insert URL. Accessed: insert date accessed.*

©CASP this work is licensed under the Creative Commons Attribution – Non-Commercial- Share A like. To view a copy of this licence, visit https://creativecommons.org/licenses/by-sa/4.0/

CASP
Critical Appraisal
Skills Programme

Study and citation: Mohammadzadeh et al. (2019) Improving emotional health and self-esteem of Malaysian Adolescents living in orphanages through Life Skills Education program: A multi-centred randomised control trial......

Section A: Is the basic study design valid for a randomised controlled trial?

**1. Did the study address a clearly focused research question?**<br>*CONSIDER:*<br>☐ *Was the study designed to assess the outcomes of an intervention?*<br>☐ *Is the research question 'focused' in terms of:*<br>• *Population studied*<br>• *Intervention given*<br>• *Comparator chosen*<br>• *Outcomes measured?*	Yes ☐ No ☐ Can't tell ☐<br><br>The study was designed to assess Life Skills Education (LSE) in improving emotional health and self-esteem for orphaned/ institutionalised adolescents.<br><br>The research question was focused in terms of:<br>Population: Malaysian adolescents living in orphanages<br>Intervention: Life-skills based intervention (Life Skills Education program)<br>Comparator: Control group pre-test, post-test and follow-up tests<br>Outcomes measured: Depression, anxiety, stress and self-esteem
**2. Was the assignment of participants to interventions randomised?**<br>*CONSIDER:*<br>• *How was randomisation carried out? Was the method appropriate?*<br>• *Was randomisation sufficient to eliminate systematic bias?*<br>• *Was the allocation sequence concealed from investigators and participants?*	Yes ☐ No ☐ Can't tell ☐<br><br>Participants were randomly divided into intervention and the placebo control group. Orphanages were randomly selected using Microsoft Excel software.
**3. Were all participants who entered the study accounted for at its conclusion?**<br>*CONSIDER:*<br>• *Were losses to follow-up and exclusions after randomisation accounted for?*<br>• *Were participants analysed in the study groups to which they were randomised (intention-to-treat analysis)?*<br>• *Was the study stopped early? If so, what was the reason?*	Yes ☐ No ☐ Can't tell ☐<br><br>The authors did not document attrition during the intervention period, post-test or follow-up. However, the authors used intention-to-treat strategy for missing data to determine the amount of distribution of missing values. Little's MCAR test revealed that 2.9% of the data was missing at random, and values were imputed using the expectation-maximisation method with importance resembling using SPSS 21.

Section B: Was the study methodologically sound?

	Yes	No	Can't tell
**4.**			
☐ **Were the participants 'blind' to intervention they were given?**	☐	☐	☐
☐ **Were the investigators 'blind' to the intervention they were giving to participants?**	☐	☐	☐
☐ **Were the people assessing/analysing outcome/s 'blinded'?**	☐	☐	☐

**5. Were the study groups similar at the start of the randomised controlled trial?**<br>*CONSIDER:*<br>☐ *Were the baseline characteristics of each study group (e.g. age, sex, socio-economic group) clearly set out?*<br>☐ *Were there any differences between the study groups that could affect the outcome/s?*	Yes ☐ No ☐ Can't tell ☐<br><br>Baseline characteristics were clearly set out. Authors used a Demographic Questionnaire which included socio-demographic characteristics of participants, age, gender, race, educational level, parental status and duration of living in an orphanage(s).<br><br>149 male participants (55%); 122 females (45%). A majority of the participants were residing in orphanages for more than 2 years (54.6%).

CASP
Critical Appraisal Skills Programme

**6. Apart from the experimental intervention, did each study group receive the same level of care (that is, were they treated equally)?**<br><br>*CONSIDER:*<br>☐ *Was there a clearly defined study protocol?*<br>☐ *If any additional interventions were given (e.g. tests or treatments), were they similar between the study groups?*<br>☐ *Were the follow-up intervals the same for each study group?*	Yes ☐ No ☐ Can't tell ☐<br><br>Control group received six sessions of the Communication for Behavioural Impact program for preventing dengue. Pre-test, post-test and follow-up test were performed for the control group, which was the same as the intervention group.<br><br>Educational sessions for the control group was shorter than the participants receiving intervention.

Section C: What are the results?

**7. Were the effects of intervention reported comprehensively?**<br><br>*CONSIDER:*<br>• *Was a power calculation undertaken?*<br>• *What outcomes were measured, and were they clearly specified?*<br>• *How were the results expressed? For binary outcomes, were relative and absolute effects reported?*<br>• *Were the results reported for each outcome in each study group at each follow-up interval?*<br>• *Was there any missing or incomplete data?*<br>• *Was there differential drop-out between the study groups that could affect the results?*<br>• *Were potential sources of bias identified?*<br>• *Which statistical tests were used?*<br>• *Were p values reported?*	Yes ☐ No ☐ Can't tell ☐<br>Effects of intervention were reported comprehensively using clear tables and diagrams. Tables compare the holistic mean difference between intervention and control groups at pre-test, post-test and follow-up test for depression, anxiety, stress and self-esteem variables. Author also compared the differences in the mean scores between variables between time points in the intervention and control groups, as well as descriptive statistics of emotional problems and self-esteem scores at three different time points for intervention and control group.<br><br>There were 2.9% of missing data in the study but the author concluded that they were missing at random after performing the Little MCAR test.<br><br>The authors conducted ANOVA within- and between-subjects effects for interventions effects for emotional problems and self-esteem. A post hoc test (Bonferroni test between groups) was applied to compare the mean scores of variables.<br><br>The level of significance (P-value) was reported at 0.05 and 0.02 (0.05/3) for adjusted P-value
**8. Was the precision of the estimate of the intervention or treatment effect reported?**<br><br>*CONSIDER:*<br>• *Were confidence intervals (CIs) reported?*	Yes ☐ No ☐ Can't tell ☐<br><br>Confidence intervals were reported and the CI was 95%.
**9. Do the benefits of the experimental intervention outweigh the harms and costs?**<br><br>*CONSIDER:*<br>☐ *What was the size of the intervention or treatment effect?*<br>☐ *Were harms or unintended effects reported for each study group?*<br>☐ *Was a cost-effectiveness analysis undertaken? (Cost-effectiveness analysis allows a comparison to be made between different interventions used in the care of the same condition or problem.)*	Yes ☐ No ☐ Can't tell ☐<br><br>The results of the post hoc test revealed a significant difference between pre-test and post-test for depression, anxiety, stress and self-esteem, with a large effect size in the intervention group.<br><br>Harms or unintended effects were not reported. Cost-effectiveness analysis was not undertaken.

Critical Appraisal Skills Programme

Section D: Will the results help locally?

10.	**Can the results be applied to your local population/in your context?**<br><br>*CONSIDER:*<br>• *Are the study participants similar to the people in your care?*<br>• *Would any differences between your population and the study participants alter the outcomes reported in the study?*<br>• *Are the outcomes important to your population?*<br>• *Are there any outcomes you would have wanted information on that have not been studied or reported?*<br>• *Are there any limitations of the study that would affect your decision?*	Yes ☐ No ☐ Can't tell ☐<br><br>These results can definitely be applied to the local population. The findings of the study could be used by local Malaysian educational planners and educational managers to design and implement continuous programs based on Life Skills Education for institutionalised and even non-institutionalised Malaysian children and adolescents to improve public health in the country.<br><br>One limitation of the study that would affect the current systematic review is that the study instruments assessing depression, anxiety, stress and self-esteem were self-administered. Thus, there could be potential social desirability bias or misunderstanding which challenges the validity of the findings.
11.	**Would the experimental intervention provide greater value to the people in your care than any of the existing interventions?**<br><br>*CONSIDER:*<br>☐ *What resources are needed to introduce this intervention taking into account time, finances, and skills development or training needs?*<br>☐ *Are you able to disinvest resources in one or more existing interventions in order to be able to re-invest in the new intervention?*	Yes ☐ No ☐ Can't tell ☐<br><br>Authors noted that Life Skills Education is a cost-effective program and easy to administer by local trainers without requiring specific tools.

**APPRAISAL SUMMARY:** *Record key points from your critical appraisal in this box. What is your conclusion about the paper? Would you use it to change your practice or to recommend changes to care/interventions used by your organisation? Could you judiciously implement this intervention without delay?*
Good rationale and research question. In-depth and comprehensive data collection, ethical consideration, data analysis and discussion. Life Skills Education program can be applied to local populations and can inform policy and practice in Western countries for youth homelessness as this program is based on life skills.<br><br>The Life Skills program was developed by the World Health Organisation through a consultation with WHO and UNICEF experts in the study field, and would have no doubt that this could be implemented in future practice without delay considering LSE's cost-effectiveness and easy administration by local trainers without using specific tools. For future researches on the effectiveness of LSE for emotional problems and self-esteem among adolescents in orphanages, self-administered questionnaires on depression, anxiety, stress and self-esteem should be re-evaluated. This will serve as a means of assessing the reliability of the participants' reported emotional problems.

www.casp-uk.net
info@casp-uk.net
Summertown Pavilion, Middle Way Oxford OX2 7LG

CASP Checklist: 11 questions to help you make sense of a **Case Control Study**

How to use this appraisal tool: Three broad issues need to be considered when appraising a case control study:

- Are the results of the study valid? (Section A)
- What are the results? (Section B)
- Will the results help locally? (Section C)

The 11 questions on the following pages are designed to help you think about these issues systematically. The first three questions are screening questions and can be answered quickly. If the answer to both is "yes", it is worth proceeding with the remaining questions. There is some degree of overlap between the questions, you are asked to record a "yes", "no" or "can't tell" to most of the questions. A number of italicised prompts are given after each question. These are designed to remind you why the question is important. Record your reasons for your answers in the spaces provided.

About: These checklists were designed to be used as educational pedagogic tools, as part of a workshop setting, therefore we do not suggest a scoring system. The core CASP checklists (randomised controlled trial & systematic review) were based on JAMA 'Users' guides to the medical literature 1994 (adapted from Guyatt GH, Sackett DL, and Cook DJ), and piloted with health care practitioners.

For each new checklist, a group of experts were assembled to develop and pilot the checklist and the workshop format with which it would be used. Over the years overall adjustments have been made to the format, but a recent survey of checklist users reiterated that the basic format continues to be useful and appropriate.

Referencing: we recommend using the Harvard style citation, i.e.: *Critical Appraisal Skills Programme (2018). CASP (insert name of checklist i.e. Case Control Study) Checklist. [online] Available at: URL. Accessed: Date Accessed.*

©CASP this work is licensed under the Creative Commons Attribution – Non-Commercial-Share A like. To view a copy of this license, visit http://creativecommons.org/licenses/by-nc-sa/3.0/ www.casp-uk.net

CASP
Critical Appraisal
Skills Programme

Paper for appraisal and reference: Noh (2018) The effect of a resilience enhancement programme for female runaway youths: A Quasi-Experimental Study

Section A: Are the results of the trial valid?

1. Did the study address a clearly focused issue?

Yes ✔
Can't Tell ☐
No ☐

HINT: An issue can be 'focused' In terms of
- the population studied
- Whether the study tried to detect a beneficial or harmful effect
- the risk factors studied

Comments:
The study addressed a clearly focused issue: The effects of a resilience enhancement program on resilience, depression, anxiety, and problem drinking among female runaway youths residing in shelters in South Korea.

2. Did the authors use an appropriate method to answer their question?

Yes ✔
Can't Tell ☐
No ☐

HINT: Consider
- Is a case control study an appropriate way of answering the question under the circumstances
- Did it address the study question

Comments:
Author used a quasi-experimental design in addressing research question. This is appropriate because author may not have full control over the assignment of participants to experimental groups in shelters; a quasi-experimental design allows for more practical approach. The study design was well suited for naturalistic environments and for studying interventions in real-world settings, and it may not be ethical to manipulate variables or assign participants randomly.

CASP Critical Appraisal Skills Programme

Is it worth continuing?

3. Were the cases recruited in an acceptable way?

Yes ✔
Can't Tell ☐
No ☐

HINT: We are looking for selection bias which might compromise validity of the findings
- are the cases defined precisely
- were the cases representative of a defined population (geographically and/or temporally)
- was there an established reliable system for selecting all the cases
- are they incident or prevalent
- is there something special about the cases
- is the time frame of the study relevant to disease/exposure
- was there a sufficient number of cases selected
- was there a power calculation

Comments:

Participants were recruited from five shelters for female runaways youths in South Korea and were representative of this population. Participants were recruited after the principal investigator (PI) explained the aim of the research to shelter residents.

Ethical considerations were reported. Both experimental and control participants were given gift certificates upon completing all three rounds of data collection.

Sample size was calculated using G*Power version 3.1.3. The study needed to obtain 80% statistical power for repeated measures with an alpha level of 0.05 and an effect size of 0.25. The estimated sample needed was 28 and the sample size of the study was n = 32, thus, the sample size was considered sufficient for the study.

4. Were the controls selected in an acceptable way?

Yes ✔
Can't Tell ☐
No ☐

HINT: We are looking for selection bias which might compromise the generalisability of the findings
- were the controls representative of the defined population (geographically and/or temporally)
- was there something special about the controls
- was the non-response high, could non-respondents be different in any way
- are they matched, population based or randomly selected
- was there a sufficient number of controls selected

Comments:

Controls were matched: They were also runaway adolescents living in shelters; therefore, representative of the defined population. PI recruited equal participants for the intervention group (n = 16) and control group (n = 16).

Overall sample size for the study was sufficient and was calculated using G*Power version 3.1.3.

CASP
Critical Appraisal Skills Programme

5. Was the exposure accurately measured to minimise bias?

Yes	☐
Can't Tell	✔
No	☐

HINT: We are looking for measurement, recall or classification bias

- was the exposure clearly defined and accurately measured
- did the authors use subjective or objective measurements
- do the measures truly reflect what they are supposed to measure (have they been validated)
- were the measurement methods similar in the cases and controls
- did the study incorporate blinding where feasible
- is the temporal relation correct (does the exposure of interest precede the outcome)

Comments:

Author used subjective measures by using self-administered questionnaires on dependant variables (resilience, depression, anxiety, and problem drinking) and background variables (age, education level, family socioeconomic status, number of runaway episodes; the amount of time spend not at home or in a shelter, length of residence time in the current shelter, and family dunction.

Measures used Korean instruments and translations of Western instruments. This reduced minsunderstanding and misinterpretation among participants.

6. (a) Aside from the experimental intervention, were the groups treated equally?

HINT: List the ones you think might be important, that the author may have missed

- genetic
- environmental
- socio-economic

List:

Groups were treated equally. At baseline, the experimental group and control group did not differ in general characteristics and family function. Both groups were assessed at pre-test, post-test and at a one-month follow-up.

6. (b) Have the authors taken account of the potential confounding factors in the design and/or in their analysis?

Yes	✔
Can't Tell	☐
No	☐

HINT: Look for

- restriction in design, and techniques e.g. modelling, stratified-, regression-, or sensitivity analysis to correct, control or adjust for confounding factors

Comments:

A potential confounding factor the author noted was that since the control and experimental group were living in the same shelter, intervention participants might disseminate the contents of the programme to the control participants. However, to avoid treatment contamination between experimental and control participants, the study employed a non-equivalent control group non-synchronised design, in which data collection for the control group was conducted and completed prior to the experimental group.

Section B: What are the results?

7. How large was the treatment effect?

HINT: Consider
- what are the bottom line results
- is the analysis appropriate to the design
- how strong is the association between exposure and outcome (look at the odds ratio)
- are the results adjusted for confounding, and might confounding still explain the association
- has adjustment made a big difference to the OR

Comments:

The analysis was appropriate to discuss research question. Significant group-by-time interaction effects for resilience, anxiety, and problem drinking at one-month follow-up. Significant decrease in depression for experimental participants occurred during the one-month intervention period, but not during the overall study period, because decreases in depression over the study period occurred for both control and experimental participants,

8. How precise was the estimate of the treatment effect?

HINT: Consider
- size of the p-value
- size of the confidence intervals
- have the authors considered all the important variables
- how was the effect of subjects refusing to participate evaluated

Comments:

PI did not document confidence intervals.

P value for resilience between pre-test and both post-test was 0.002 and 0.007 at a one-month follow-up. Depression at pre-test and post-test was p = 0.037. Anxiety at pre-, post- and follow-up test was p = 0.022. For problem drinking, p value was p = 0.001 at pre- and post-test and 0.038 at follow-up. The size of p values across variables and interval times provided evidence in support of a true intervention effect.

CASP
Critical Appraisal
Skills Programme

9. Do you believe the results?

Yes ✔
No ☐

HINT: Consider
- big effect is hard to ignore!
- Can it be due to chance, bias, or confounding
- are the design and methods of this study sufficiently flawed to make the results unreliable
- consider Bradford Hills criteria (e.g. time sequence, does-response gradient, strength, biological plausibility)

Comments:
The small p-value suggests that the Resilience Enhancement Programme on dependant variables was unlikely to occur by random chance alone.

Section C: Will the results help locally?

10. Can the results be applied to the local population?

Yes ✔
Can't Tell ☐
No ☐

HINT: Consider whether
- the subjects covered in the study could be sufficiently different from your population to cause concern
- your local setting is likely to differ much from that of the study
- can you quantify the local benefits and harms

Comments:
The Resilient Enhancement Programme developed by the PI can be used locally for South Korean runaway adolescents. Although these are just preliminary results, these findings will help inform future researches and practice.

11. Do the results of this study fit with other available evidence?

Yes ✔
Can't Tell ☐
No ☐

HINT: Consider
- all the available evidence from RCT's Systematic Reviews, Cohort Studies, and Case Control Studies as well, for consistency

Comments:
The results for the study informs future research and practice in terms of the differences in mental health status for female runaway adolescents. These results further contributes to and enhance existing literature on the characteristics and risks associated with female runaway youth.

Remember One observational study rarely provides sufficiently robust evidence to recommend changes to clinical practice or within health policy decision making. However, for certain questions observational studies provide the only evidence. Recommendations from observational studies are always stronger when supported by other evidence.

CASP Randomised Controlled Trial Standard Checklist:
11 questions to help you make sense of a randomised controlled trial (RCT)

Main issues for consideration: Several aspects need to be considered when appraising a randomised controlled trial:

- Is the basic study design valid for a randomised controlled trial? (Section A)
- Was the study methodologically sound? (Section B)
- What are the results? (Section C)
- Will the results help locally? (Section D)

The 11 questions in the checklist are designed to help you think about these aspects systematically.

How to use this appraisal tool: The first three questions (Section A) are screening questions about the validity of the basic study design and can be answered quickly. If, in light of your responses to Section A, you think the study design is valid, continue to Section B to assess whether the study was methodologically sound and if it is worth continuing with the appraisal by answering the remaining questions in Sections C and D.

Record 'Yes', 'No' or 'Can't tell' in response to the questions. Prompts below all but one of the questions highlight the issues it is important to consider. Record the reasons for your answers in the space provided. As CASP checklists were designed to be used as educational/teaching tools in a workshop setting, we do not recommend using a scoring system.

About CASP Checklists: The CASP RCT checklist was originally based on JAMA Users' guides to the medical literature 1994 (adapted from Guyatt GH, Sackett DL and Cook DJ), and piloted with healthcare practitioners. This version has been updated taking into account the CONSORT 2010 guideline (http://www.consort-statement.org/consort-2010, accessed 16 September 2020).

Citation: CASP recommends using the Harvard style, i.e., *Critical Appraisal Skills Programme (2021). CASP (insert name of checklist i.e. Randomised Controlled Trial) Checklist. [online] Available at: insert URL. Accessed: insert date accessed.*

©CASP this work is licensed under the Creative Commons Attribution – Non-Commercial- Share A like. To view a copy of this licence, visit https://creativecommons.org/licenses/by-sa/4.0/

CASP
Critical Appraisal Skills Programme

Study and citation: Noh & Choi (2020) Development of a Family-Based Mental Health Program for Runaway Adolescents Using an Intervention Mapping Protocol

Section A: Is the basic study design valid for a randomised controlled trial?

**1. Did the study address a clearly focused research question?**<br>*CONSIDER:*<br>☐ *Was the study designed to assess the outcomes of an intervention?*<br>☐ *Is the research question 'focused' in terms of:*<br>• *Population studied*<br>• *Intervention given*<br>• *Comparator chosen*<br>• *Outcomes measured?*	Yes ☐ No ☐ Can't tell ☐<br>The authors developed a mental health program and planned to use a RCT to evaluate the effects of the program on improving adolescents' mental health status and perceived family functioning. An RCT was not and has not been conducted to evaluate the program, up to this present day. However, interviews with adolescents living in shelters were conducted using purposive sampling so they could develop a program based on their lived experiences.<br><br>The research question was focused in terms of:<br>Population: Runaway adolescents<br>Intervention: Family-based mental health program<br>Comparators: Comparison group once intervention has been implemented<br>Outcomes measured: Behavioural outcomes and environmental outcomes
**2. Was the assignment of participants to interventions randomised?**<br>*CONSIDER:*<br>• *How was randomisation carried out? Was the method appropriate?*<br>• *Was randomisation sufficient to eliminate systematic bias?*<br>• *Was the allocation sequence concealed from investigators and participants?*	Yes ☐ No ☐ Can't tell ☐<br>Researchers plan to divide experimental and control groups via a computer-generated random allocation after they conduct an RCT to evaluate the program.
**3. Were all participants who entered the study accounted for at its conclusion?**<br>*CONSIDER:*<br>• *Were losses to follow-up and exclusions after randomisation accounted for?*<br>• *Were participants analysed in the study groups to which they were randomised (intention-to-treat analysis)?*<br>• *Was the study stopped early? If so, what was the reason?*	Yes ☐ No ☐ Can't tell ☐<br>Program not yet evaluated.

Section B: Was the study methodologically sound?

**4.**<br>☐ **Were the participants 'blind' to intervention they were given?**<br>☐ **Were the investigators 'blind' to the intervention they were giving to participants?**<br>☐ **Were the people assessing/analysing outcome/s 'blinded'?**	Yes No Can't tell<br>☐ ☐ ☐<br>☐ ☐ ☐<br>☐ ☐ ☐
**5. Were the study groups similar at the start of the randomised controlled trial?**<br>*CONSIDER:*<br>☐ *Were the baseline characteristics of each study group (e.g. age, sex, socio-economic group) clearly set out?*<br>☐ *Were there any differences between the study groups that could affect the outcome/s?*	Yes ☐ No ☐ Can't tell ☐<br>Despite an absence of an RCT to evaluate the program, the authors specified the baseline characteristics they plan to recruit for participants, as well as the exclusion criteria.

**6. Apart from the experimental intervention, did each study group receive the same level of care (that is, were they treated equally)?**<br><br>*CONSIDER:*<br>☐ *Was there a clearly defined study protocol?*<br>☐ *If any additional interventions were given (e.g. tests or treatments), were they similar between the study groups?*<br>☐ *Were the follow-up intervals the same for each study group?*	Yes ☐ No ☐ Can't tell ☐<br><br>Program not yet evaluated. However, the researchers plan to collect participants' data using self-administered questionnaire surveys. Assessment time points will include baseline, immediately after the program and one month after program completion.

Section C: What are the results?

**7. Were the effects of intervention reported comprehensively?**<br><br>*CONSIDER:*<br>• *Was a power calculation undertaken?*<br>• *What outcomes were measured, and were they clearly specified?*<br>• *How were the results expressed? For binary outcomes, were relative and absolute effects reported?*<br>• *Were the results reported for each outcome in each study group at each follow-up interval?*<br>• *Was there any missing or incomplete data?*<br>• *Was there differential drop-out between the study groups that could affect the results?*<br>• *Were potential sources of bias identified?*<br>• *Which statistical tests were used?*<br>• *Were p values reported?*	Yes ☐ No ☐ Can't tell ☐<br><br>Program not yet evaluated to test its effectiveness. No data present in this paper. Statistical outcomes were not clearly specified.<br><br>Researchers plan to analyse future data using IBM SPSS, version 26.0.<br><br>Intervention effects will be examined using a one-way repeated measures MANOVA.<br><br>A power analysis conducted using the G* Power program indicated that a total sample of 211 subjects would be needed to detect a medium effect (f = 0.25) with 80% power using MANOVA at an alpha level 0.05. Therefore, researchers will collect data from n = 236 participants (experimental: n = 118; comparison: n = 188) to accommodate the expected attrition of 10% over the three-month period from the baseline assessment to the final evaluation.
**8. Was the precision of the estimate of the intervention or treatment effect reported?**<br><br>*CONSIDER:*<br>• *Were confidence intervals (CIs) reported?*	Yes ☐ No ☐ Can't tell ☐<br><br>Program not yet evaluated to test its effectiveness. Therefore, CI has not been reported.
**9. Do the benefits of the experimental intervention outweigh the harms and costs?**<br><br>*CONSIDER:*<br>☐ *What was the size of the intervention or treatment effect?*<br>☐ *Were harms or unintended effects reported for each study group?*<br>☐ *Was a cost-effectiveness analysis undertaken? (Cost-effectiveness analysis allows a comparison to be made between different interventions used in the care of the same condition or problem.)*	Yes ☐ No ☐ Can't tell ☐<br><br>Program not yet evaluated to test its effectiveness; no data present to see the size of intervention of treatment effect.

CASP
Critical Appraisal Skills Programme

Section D: Will the results help locally?

**10.**	**Can the results be applied to your local population/in your context?**<br><br>*CONSIDER:*<br>• *Are the study participants similar to the people in your care?*<br>• *Would any differences between your population and the study participants alter the outcomes reported in the study?*<br>• *Are the outcomes important to your population?*<br>• *Are there any outcomes you would have wanted information on that have not been studied or reported?*<br>• *Are there any limitations of the study that would affect your decision?*	Yes ☐ No ☐ Can't tell ☐<br><br>The development of a family-based mental health program for runaway adolescents can be applied to local populations (South Korea) as there was an emphasis on involving families during interventions.
**11.**	**Would the experimental intervention provide greater value to the people in your care than any of the existing interventions?**<br><br>*CONSIDER:*<br>☐ *What resources are needed to introduce this intervention taking into account time, finances, and skills development or training needs?*<br>☐ *Are you able to disinvest resources in one or more existing interventions in order to be able to re-invest in the new intervention?*	Yes ☐ No ☐ Can't tell ☐<br><br>This program would require a lot of multidisciplinary collaboration and training. It would also require some modification as the program was developed by interviewing the lived experiences of sheltered adolescents in South Korea.

APPRAISAL SUMMARY: *Record key points from your critical appraisal in this box. What is your conclusion about the paper? Would you use it to change your practice or to recommend changes to care/interventions used by your organisation? Could you judiciously implement this intervention without delay?*

Good rationale and basis for the development of a program. Comprehensive program development. Focused on the lived experiences of South Korean adolescents living in shelters. Strong focus on family.

However, data on the outcomes of intervention effect would have been beneficial in providing insight into whether the program is achieving its intended goals. This would enable informed decision-making regarding the continuation/modification/termination of the program. Outcomes could also contribute to evidence-based practice. The authors of the paper should evaluate the effectiveness of the program in the near future.

CASP
Critical Appraisal
Skills Programme

www.casp-uk.net
info@casp-uk.net
Summertown Pavilion, Middle
Way Oxford OX2 7LG

CASP Checklist: 12 questions to help you make sense of a **Cohort Study**

How to use this appraisal tool: Three broad issues need to be considered when appraising a cohort study:

- Are the results of the study valid? (Section A)
- What are the results? (Section B)
- Will the results help locally? (Section C)

The 12 questions on the following pages are designed to help you think about these issues systematically. The first two questions are screening questions and can be answered quickly. If the answer to both is "yes", it is worth proceeding with the remaining questions. There is some degree of overlap between the questions, you are asked to record a "yes", "no" or "can't tell" to most of the questions. A number of italicised prompts are given after each question. These are designed to remind you why the question is important. Record your reasons for your answers in the spaces provided.

About: These checklists were designed to be used as educational pedagogic tools, as part of a workshop setting, therefore we do not suggest a scoring system. The core CASP checklists (randomised controlled trial & systematic review) were based on JAMA 'Users' guides to the medical literature 1994 (adapted from Guyatt GH, Sackett DL, and Cook DJ), and piloted with health care practitioners.

For each new checklist, a group of experts were assembled to develop and pilot the checklist and the workshop format with which it would be used. Over the years overall adjustments have been made to the format, but a recent survey of checklist users reiterated that the basic format continues to be useful and appropriate.

Referencing: we recommend using the Harvard style citation, i.e.: *Critical Appraisal Skills Programme (2018). CASP (insert name of checklist i.e. Cohort Study) Checklist. [online] Available at: URL. Accessed: Date Accessed.*

©CASP this work is licensed under the Creative Commons Attribution – Non-Commercial-Share A like. To view a copy of this license, visit http://creativecommons.org/licenses/by-nc-sa/3.0/ www.casp-uk.net

CASP
Critical Appraisal Skills Programme

Paper for appraisal and reference: Sarmini & Sukartiningsih (2018) From the Road to the Arena: The Role of Kampung Anak Negeri for Street Children ....

Section A: Are the results of the study valid?

1. Did the study address a clearly focused issue?

Yes	✔
Can't Tell	
No	

HINT: A question can be 'focused' in terms of
- the population studied
- the risk factors studied
- is it clear whether the study tried to detect a beneficial or harmful effect
- the outcomes considered

Comments:

Research question focused in terms of population studied (street children) and the risks associated with being a street child. Findings are valid and explored the beneficial role of the program 'Kampung Anak Negeri' (KAN) in helping street children.

2. Was the cohort recruited in an acceptable way?

Yes	✔
Can't Tell	
No	

HINT: Look for selection bias which might compromise the generalisability of the findings:
- was the cohort representative of a defined population
- was there something special about the cohort
- was everybody included who should have been

Comments:

Cohort were representative of the focused population. Participants in the study were street children who were placed in the Children's Village.

Is it worth continuing?

CASP
Critical Appraisal
Skills Programme

3. Was the exposure accurately measured to minimise bias?

Yes ✔
Can't Tell ☐
No ☐

HINT: Look for measurement or classification bias:
- did they use subjective or objective measurements
- do the measurements truly reflect what you want them to (have they been validated)
- were all the subjects classified into exposure groups using the same procedure

Comments:
The 'exposure' in this study was the involvement or participation of street children in KAN. Data collection used both subjective and objective measurements: observation methods and in-depth interviews.

These reflect the role of KAN, which answers the research question.

4. Was the outcome accurately measured to minimise bias?

Yes ☐
Can't Tell ✔
No ☐

HINT: Look for measurement or classification bias:
- did they use subjective or objective measurements
- do the measurements truly reflect what you want them to (have they been validated)
- has a reliable system been established for detecting all the cases (for measuring disease occurrence)
- were the measurement methods similar in the different groups
- were the subjects and/or the outcome assessor blinded to exposure (does this matter)

Comments:

The role of this paper was to explore the five roles of KAN and the authors did not assess or validate the program's effectiveness through measurement.

CASP
Critical Appraisal
Skills Programme

5. (a) Have the authors identified all important confounding factors?

Yes ☐
Can't Tell ☐
No ☑

HINT:
- list the ones you think might be important, and ones the author missed

Comments:
Authors did not identify any confounding variables.

Possible confounding variables could be selection bias. Although participants were street children in the children's village, there could be different baseline characteristics between participants who were observed/interviewed and those who were not. These were not accounted for.

5. (b) Have they taken account of the confounding factors in the design and/or analysis?

Yes ☐
Can't Tell ☑
No ☐

HINT:
- look for restriction in design, and techniques e.g. modelling, stratified-, regression-, or sensitivity analysis to correct, control or adjust for confounding factors

Comments:
Participants were collected purposively. Participant characteristics (e.g., age, sex) were not documented by the authors.

The authors used Talcott Parsons functional structural theory perspective to analyse data. Analysis had four stages: data collection, data reduction, data presentation and conclusion verification.

Analysis may have subjectivity and bias as it relies on researcher's interpretation.

6. (a) Was the follow up of subjects complete enough?

Yes ☐
Can't Tell ☐
No ☑

HINT: Consider
- the good or bad effects should have had long enough to reveal themselves
- the persons that are lost to follow-up may have different outcomes than those available for assessment
- in an open or dynamic cohort, was there anything special about the outcome of the people leaving, or the exposure of the people entering the cohort

6. (b) Was the follow up of subjects long enough?

Yes ☐
Can't Tell ☐
No ☑

CASP
Critical Appraisal
Skills Programme

Comments:

This paper was merely exploring the role of KAN. The authors did not follow up on the children post-KAN to see if the program had lasting effects.

Section B: What are the results?

7. What are the results of this study?

HINT: Consider
- what are the bottom line results
- have they reported the rate or the proportion between the exposed/unexposed, the ratio/rate difference
- how strong is the association between exposure and outcome (RR)
- what is the absolute risk reduction (ARR)

Comments:

The findings of this study explored the role of KAN in facilitating the transition of street children towards a regular life.

8. How precise are the results?

HINT:
- look for the range of the confidence intervals, if given

Comments:

This paper was a qualitative case study and did not need statistical analysis.

CASP
Critical Appraisal
Skills Programme

9. Do you believe the results?

Yes ✔
Can't Tell ☐
No ☐

HINT: Consider
- big effect is hard to ignore
- can it be due to bias, chance or confounding
- are the design and methods of this study sufficiently flawed to make the results unreliable
- Bradford Hills criteria (e.g. time sequence, dose-response gradient, biological plausibility, consistency)

Comments:
Findings of the study explore the role of KAN and the authors wrote a comprehensive paper exploring the six roles and responsibilities of the KAN.

Section C: Will the results help locally?

10. Can the results be applied to the local population?

Yes ✔
Can't Tell ☐
No ☐

HINT: Consider whether
- a cohort study was the appropriate method to answer this question
- the subjects covered in this study could be sufficiently different from your population to cause concern
- your local setting is likely to differ much from that of the study
- you can quantify the local benefits and harms

Comments:
Findings will be able to help locally.

11. Do the results of this study fit with other available evidence?

Yes ✔
Can't Tell ☐
No ☐

Comments:
Results from the paper contributes to and enhance existing literature on the programs for street children.

12. What are the implications of this study for practice?

Yes ☑ Can't Tell ☐ No ☐

HINT: Consider
- one observational study rarely provides sufficiently robust evidence to recommend changes to clinical practice or within health policy decision making
- for certain questions, observational studies provide the only evidence
- recommendations from observational studies are always stronger when supported by other evidence

Comments:
Overall, KAN serves as a comprehensive support system for street children that respects and integrates Indonesian culture into its program. This underscored the importance of cultural contexts for supporting street children and has the potential to guide policy-making and implementation.

CASP
Critical Appraisal Skills Programme

www.casp-uk.net
info@casp-uk.net
Summertown Pavilion, Middle Way Oxford OX2 7LG

CASP Checklist: 10 questions to help you make sense of a **Qualitative** research

How to use this appraisal tool: Three broad issues need to be considered when appraising a qualitative study:

- Are the results of the study valid? (Section A)
- What are the results? (Section B)
- Will the results help locally? (Section C)

The 10 questions on the following pages are designed to help you think about these issues systematically. The first two questions are screening questions and can be answered quickly. If the answer to both is "yes", it is worth proceeding with the remaining questions. There is some degree of overlap between the questions, you are asked to record a "yes", "no" or "can't tell" to most of the questions. A number of italicised prompts are given after each question. These are designed to remind you why the question is important. Record your reasons for your answers in the spaces provided.

About: These checklists were designed to be used as educational pedagogic tools, as part of a workshop setting, therefore we do not suggest a scoring system. The core CASP checklists (randomised controlled trial & systematic review) were based on JAMA 'Users' guides to the medical literature 1994 (adapted from Guyatt GH, Sackett DL, and Cook DJ), and piloted with health care practitioners.

For each new checklist, a group of experts were assembled to develop and pilot the checklist and the workshop format with which it would be used. Over the years overall adjustments have been made to the format, but a recent survey of checklist users reiterated that the basic format continues to be useful and appropriate.

Referencing: we recommend using the Harvard style citation, i.e.: *Critical Appraisal Skills Programme (2018). CASP (insert name of checklist i.e. Qualitative) Checklist. [online] Available at: URL. Accessed: Date Accessed.*

©CASP this work is licensed under the Creative Commons Attribution – Non-Commercial-Share A like. To view a copy of this license, visit http://creativecommons.org/licenses/by-nc-sa/3.0/ www.casp-uk.net

CASP
Critical Appraisal Skills Programme

Paper for appraisal and reference: Solong et al. (2023) Street Child Management Policy at Social Office of Makassar City, Indonesia

Section A: Are the results valid?

1. Was there a clear statement of the aims of the research?

Yes ✔ | Can't Tell ☐ | No ☐

HINT: Consider
- what was the goal of the research
- why it was thought important
- its relevance

Comments:
The paper addressed clear aims for the research and the research question was focused. The purpose of the research was to find out the policies implemented by the government in supporting street children.

This research was crucial to shed light on the social welfare of street children, human rights, public health, education and social integration.

2. Is a qualitative methodology appropriate?

Yes ✔ | Can't Tell ☐ | No ☐

HINT: Consider
- If the research seeks to interpret or illuminate the actions and/or subjective experiences of research participants
- Is qualitative research the right methodology for addressing the research goal

Comments:
Qualitative methodology was appropriate for addressing the research question. This was well-suited for exploring the informants experiences and perspectives. Primary data collection such as field observations and interviews were appropriate. The qualitative methodology provides an understanding of the complexities and nuances of street children and government policies.

Is it worth continuing?

3. Was the research design appropriate to address the aims of the research?

Yes ✔ | Can't Tell ☐ | No ☐

HINT: Consider
- if the researcher has justified the research design (e.g. have they discussed how they decided which method to use)

Comments:

By combining field observations and interviews with key informants, the research design allows for a comprehensive exploration of the policies implemented by the government to support street children.

CASP Critical Appraisal Skills Programme

4. Was the recruitment strategy appropriate to the aims of the research?

Yes	✔
Can't Tell	
No	

HINT: Consider
- If the researcher has explained how the participants were selected
- If they explained why the participants they selected were the most appropriate to provide access to the type of knowledge sought by the study
- If there are any discussions around recruitment (e.g. why some people chose not to take part)

Comments:
Research informants were recruited using purposive sampling. This was appropriate considering the nature of the research rationale and research question.

Purposive sampling allowed the researchers to select participants who could provide valuable insights into the street children policies, ensuring that the study's objectives could be effectively addressed. It enabled the researchers to target individuals with relevant experience and/or expertise which enhanced the quality and relevance of the data collected.

5. Was the data collected in a way that addressed the research issue?

Yes	✔
Can't Tell	
No	

HINT: Consider
- If the setting for the data collection was justified
- If it is clear how data were collected (e.g. focus group, semi-structured interview etc.)
- If the researcher has justified the methods chosen
- If the researcher has made the methods explicit (e.g. for interview method, is there an indication of how interviews are conducted, or did they use a topic guide)
- If methods were modified during the study. If so, has the researcher explained how and why
- If the form of data is clear (e.g. tape recordings, video material, notes etc.)
- If the researcher has discussed saturation of data

Comments:
Data collection approach effectively addressed the research issue of investigating the policies implemented by the government to support street children.

Observations allowed the researchers to directly observe behaviours and interaction, while in-depth interviews provided insights informants' perspectives and/or lived experiences. Researchers minimised researcher bias and remained objective in their analysis which was evident through thorough documentation of field notes and fact-checking from secondary data (e.g., photos, laws and regulations of street children field notes).

Supplementing primary data with secondary sources such as publications, books, journals, and magazines enhances the breadth and depth of the research. Secondary data can provide theoretical frameworks, historical perspectives, comparative analyses, and additional empirical evidence relevant to the research issue. It allowed the researchers to contextualise their findings within existing literature and perspectives on government policies for street children.

CASP
Critical Appraisal
Skills Programme

6. Has the relationship between researcher and participants been adequately considered?

Yes	☐
Can't Tell	☐
No	☑

HINT: Consider
- If the researcher critically examined their own role, potential bias and influence during (a) formulation of the research questions (b) data collection, including sample recruitment and choice of location
- How the researcher responded to events during the study and whether they considered the implications of any changes in the research design

Comments:

The relationship between the researcher and participants was not adequately considered; the researcher did not examine their own role.

Section B: What are the results?

7. Have ethical issues been taken into consideration?

Yes	☐
Can't Tell	☐
No	☑

HINT: Consider
- If there are sufficient details of how the research was explained to participants for the reader to assess whether ethical standards were maintained
- If the researcher has discussed issues raised by the study (e.g. issues around informed consent or confidentiality or how they have handled the effects of the study on the participants during and after the study)
- If approval has been sought from the ethics committee

Comments:

Ethical issues were not taken into consideration and were not documented in the paper. Approval was not sought out from the ethics committee.

CASP
Critical Appraisal Skills Programme

8. Was the data analysis sufficiently rigorous?

Yes ✔
Can't Tell ☐
No ☐

HINT: Consider
- If there is an in-depth description of the analysis process
- If thematic analysis is used. If so, is it clear how the categories/themes were derived from the data
- Whether the researcher explains how the data presented were selected from the original sample to demonstrate the analysis process
- If sufficient data are presented to support the findings
- To what extent contradictory data are taken into account
- Whether the researcher critically examined their own role, potential bias and influence during analysis and selection of data for presentation

Comments:
The researchers used a four-step analysis to analyse the data: Data selection, data reduction data display and drawing conclusions/verifying.

The researchers wrote an in-depth description of their data analysis techniques

9. Is there a clear statement of findings?

Yes ✔
Can't Tell ☐
No ☐

HINT: Consider whether
- If the findings are explicit
- If there is adequate discussion of the evidence both for and against the researcher's arguments
- If the researcher has discussed the credibility of their findings (e.g. triangulation, respondent validation, more than one analyst)
- If the findings are discussed in relation to the original research question

Comments:
The paper has a clear statement of findings of government policies on street children. Researchers used secondary data which provided additional sources of information and perspectives that can help verify the accuracy and reliability of primary data or claims.

CASP Critical Appraisal Skills Programme

Section C: Will the results help locally?

10. How valuable is the research?

HINT: Consider
- If the researcher discusses the contribution the study makes to existing knowledge or understanding (e.g. do they consider the findings in relation to current practice or policy, or relevant research-based literature
- If they identify new areas where research is necessary
- If the researchers have discussed whether or how the findings can be transferred to other populations or considered other ways the research may be used

Comments:

In addition to finding out the policies implemented by the government to support street children, the researchers also identified what factors are inhibiting and encouraging the government in carrying out policies to support or assist street children.

Understanding the barriers that hinder government efforts to support street children can guide policymakers in addressing gaps and weaknesses in existing policies. Knowledge of the factors influencing government actions can help policymakers allocate resources more effectively to support street children. Prioritising areas where resources are most needed and targeting interventions based on identified barriers and enablers, policymakers can maximise the impact of limited resources and ensure that interventions reach those who need them most.
